# Supplementary material for: Ratiometric afterglow luminescent nanoplatform enables reliable quantification and molecular imaging
Source: Nat Commun. 2022 Apr 25;13:2216. doi: 10.1038/s41467-022-29894-1 (PMC9039063; doi:10.1038/s41467-022-29894-1)
Supplement: Supplementary file 1 — Supplementary information [file 41467_2022_29894_MOESM1_ESM.pdf]

Ratiometric afterglow luminescent nanoplatform enables reliable quantification and molecular imaging

Yongchao Liu, Lili Teng, Yifan Lyu, Guosheng Song*, Xiao-Bing Zhang*, and Weihong Tan

Molecular Science and Biomedicine Laboratory (MBL), State Key Laboratory of Chemo/Biosensing and Chemometrics, College of Chemistry and Chemical Engineering, Hunan University, Changsha, 410082. P. R. China.

**e-mail: xbzhang@hnu.edu.cn; songgs@hnu.edu.cn*

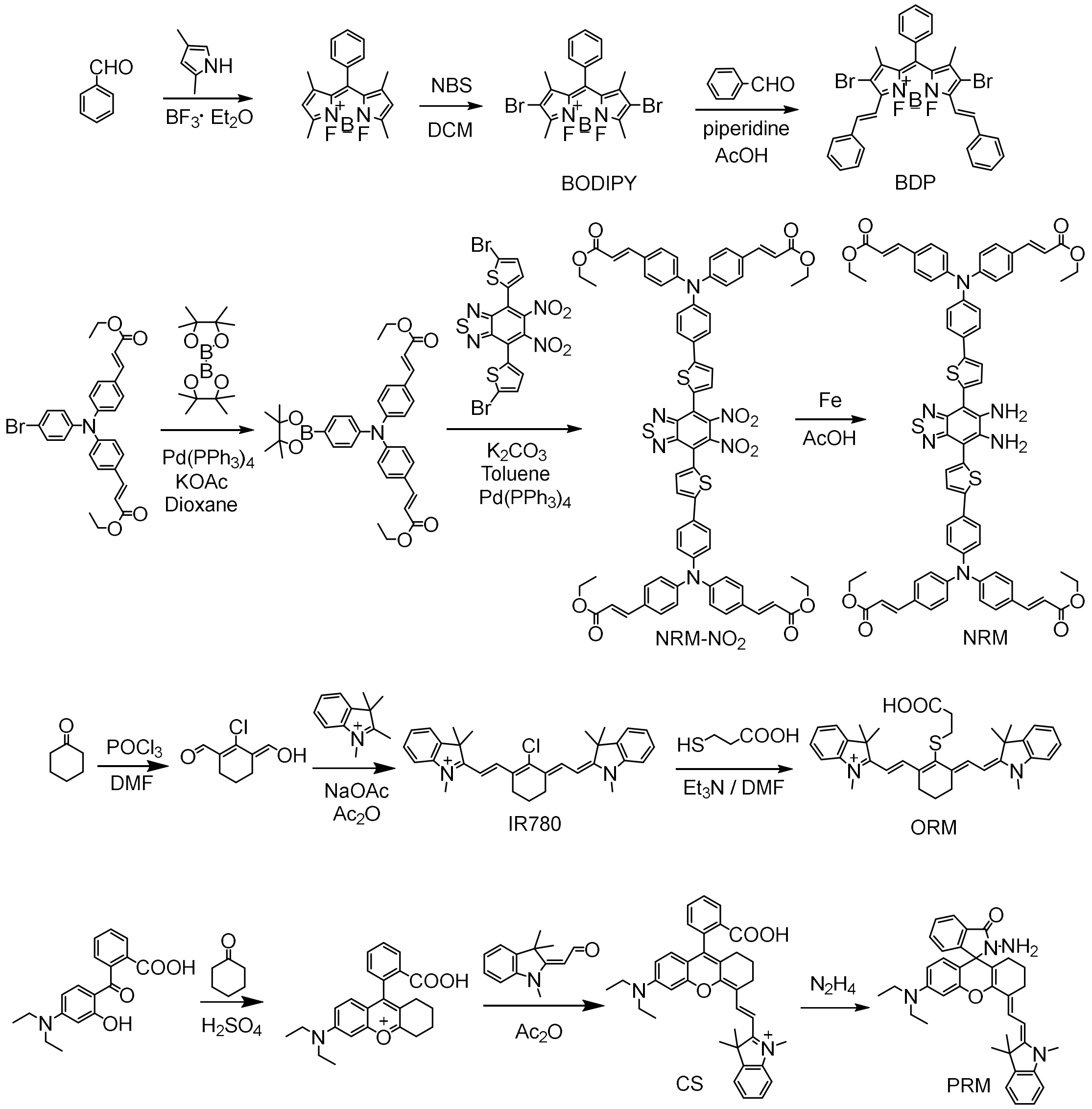

Supplementary Fig. 1. The synthetic route of BDP, NRM, ORM and PRM. The synthesis of BODIPY, NRM-NO2 and IR-780 was performed according to the literature.[1, 2, 3].

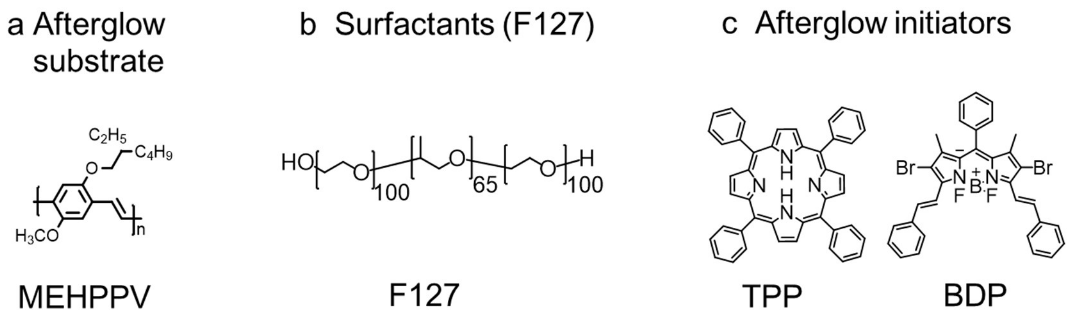

Supplementary Fig. 2 Chemical structures of each unit (afterglow substrate, afterglow initiator, surfactant, and responsive molecules).

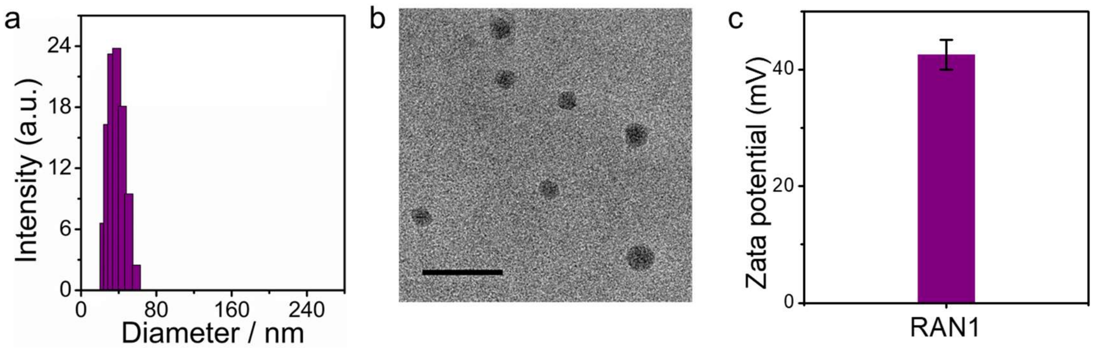

Supplementary Fig. 3 a, Dynamic light scattering (DLS), **b**, transmission electron microscopy (TEM) image and **c**, Zata potential of RAN1. Scale bar: 100 nm. Data are presented as mean values ± s.d. (n = 3).

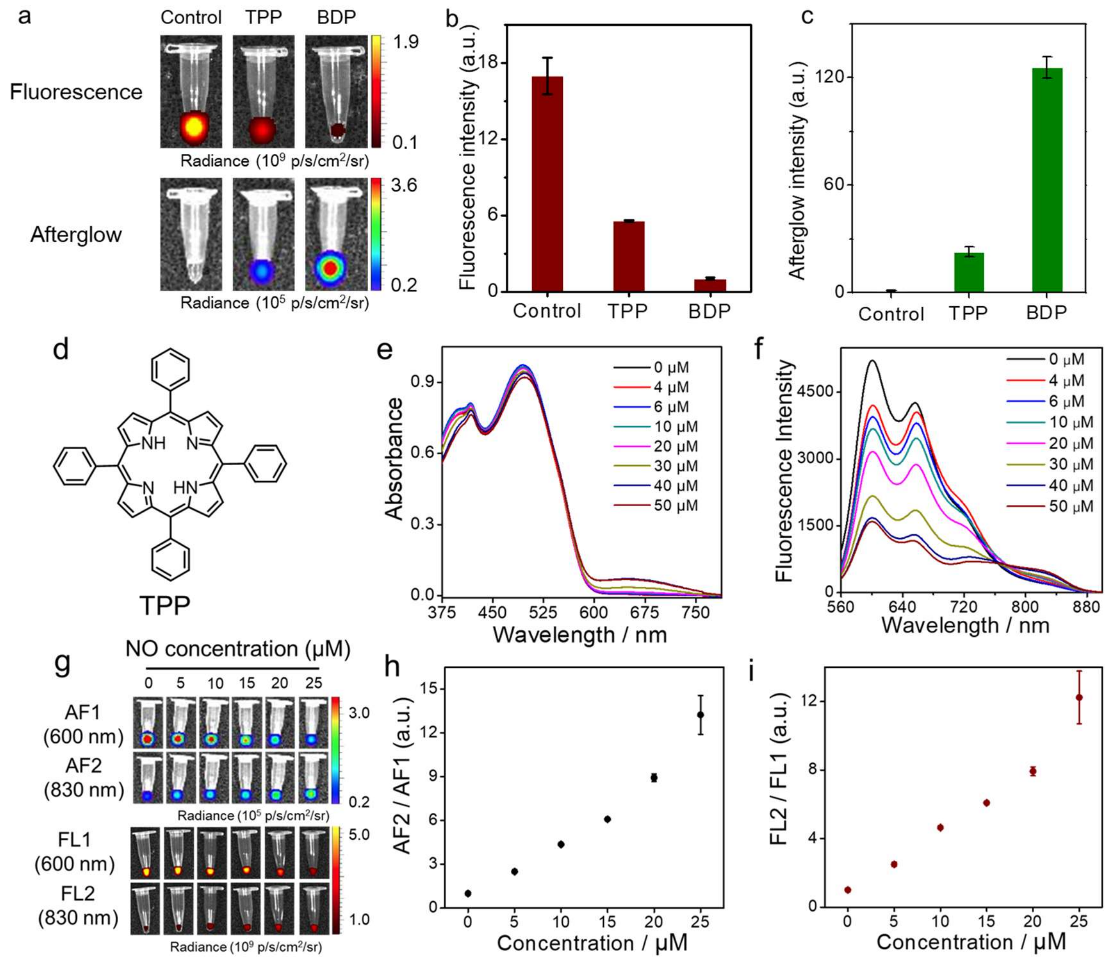

Supplementary Fig. 4 a, Fluorescent and afterglow luminescent images of only MEHPPV (25 μg/mL), TPP or BDP doped MEHPPV (25 μg/mL) nanoparticles. **b**, Quantification of fluorescence intensities and **c**, afterglow intensities of MEHPPV in (**a**). Fluorescent and afterglow images were acquired on an IVIS Spectrum imaging system equipped with a DsRed emission filter. Afterglow was initiated by 660 nm (0.80 W/cm^2) laser. **d**, Chemical structure of TPP. Normalized **e**, absorption spectra and **f**, fluorescence spectra of TPP-doped RAN1 (10 μg/mL) treated with different concentrations of NO. **g**, Afterglow luminescent images and **b**, fluorescent images of TPP-doped RAN1 (20 μg/mL) treated with different concentrations of NO. **h**, Normalized afterglow intensity ratios (AF2/AF1) and **i**, fluorescence intensity ratios (FL2/FL1) as a function of NO concentrations. Data are presented as mean values ± s.d. (n = 3). The near-infrared afterglow initiators (e.g.,

meso-tetraphenylporphyrin (TPP) and boron dipyrromethene (BDP)) were selected as $^1O_2$ generators to initiate the afterglow[4, 5, 6]. These results showed that both TPP and BDP could significantly enhance afterglow of MEHPPV after pre-irradiation of 660 nm laser, but BDP-doped RAN1 showed better NO response compared with TPP-doped RAN1.

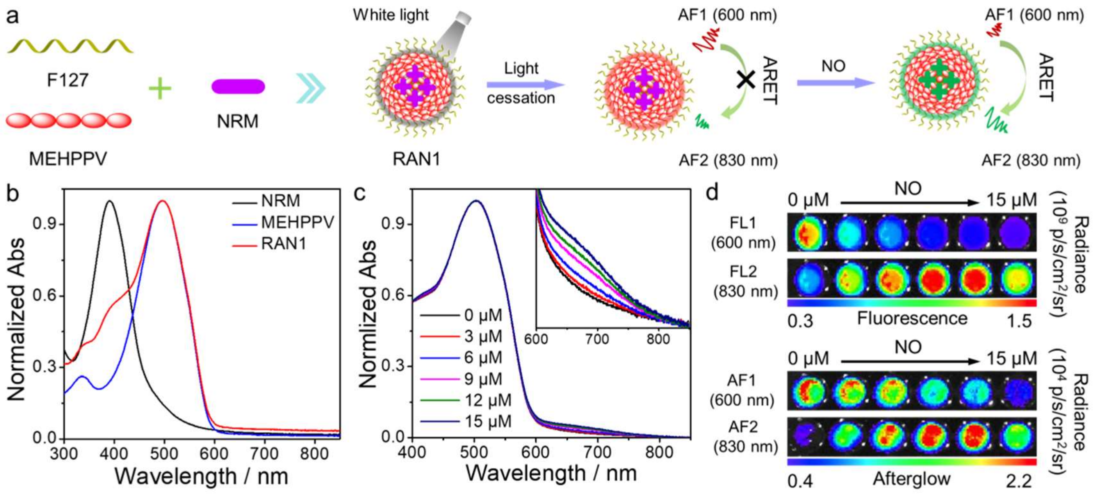

Supplementary Fig. 5 a, General strategy for synthesis and schematic illustration of RAN1 (without photosensitizer) for ratiometric afterglow sensing of NO to eliminate the influence of afterglow initiator and thus improve the sensing ability of RAN1 to NO. **b**, Normalized absorption spectra of RAN1 (10 μg/mL, without photosensitizer) and other nanoparticles. **c**, Normalized absorption spectra of RAN1 (10 μg/mL, without photosensitizer) treated with different concentrations of NO. Insert: amplification of the absorption spectra with the absorption wavelength from 600 to 850 nm. **d**, Fluorescent images and afterglow images of RAN1 (20 μg/mL, without photosensitizer) treated with different concentrations of NO. The samples were pre-illuminated with white light (0.4 W/cm^2) for 30 s.

With the enhancement of NO concentration, the nanoprobe showed enhanced absorption at 660 nm, and the fluorescent images and afterglow images collected after white light irradiation showed the decreased luminescence intensity of AF1 and increased intensity of AF2, corresponding to ARET process from AF1 to AF2, further confirming that the reaction of NRM with NO is not affected by the afterglow initiator.

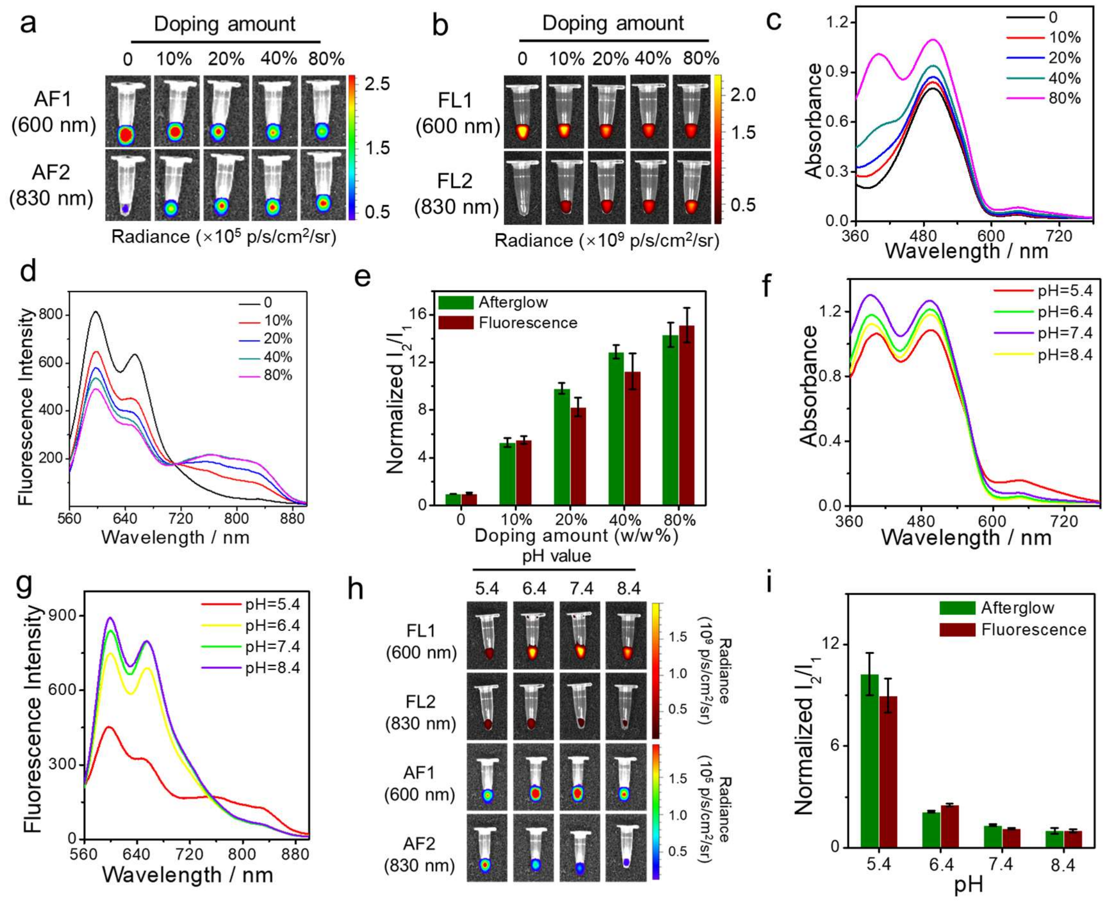

Supplementary Fig. 6 **a**, Afterglow luminescent images and **b**, fluorescent images of RAN1 (20 μg/mL) with different doping amounts of NRM. **c**, Normalized absorption spectra and **d**, fluorescence spectra of RAN1 with different doping amounts of NRM. **e**, Normalized afterglow intensity ratios (AF2/AF1) and fluorescence intensity ratios (FL2/FL1) in (**a**) and (**b**). **f**, Normalized absorption spectra and **g**, fluorescence spectra of RAN1 with different pH values. **h**. Afterglow luminescent images and fluorescent images of RAN1 (20 μg/mL) with different pH values. **i**, Normalized afterglow intensity ratios (AF2/AF1) and fluorescence intensity ratios (FL2/FL1) in (**h**), respectively. Data are presented as mean values ± s.d. (n = 3). These results showed that 80% of NRM doping amounts led to the optimal response and that the reaction of NRM and NO is induced by acid.

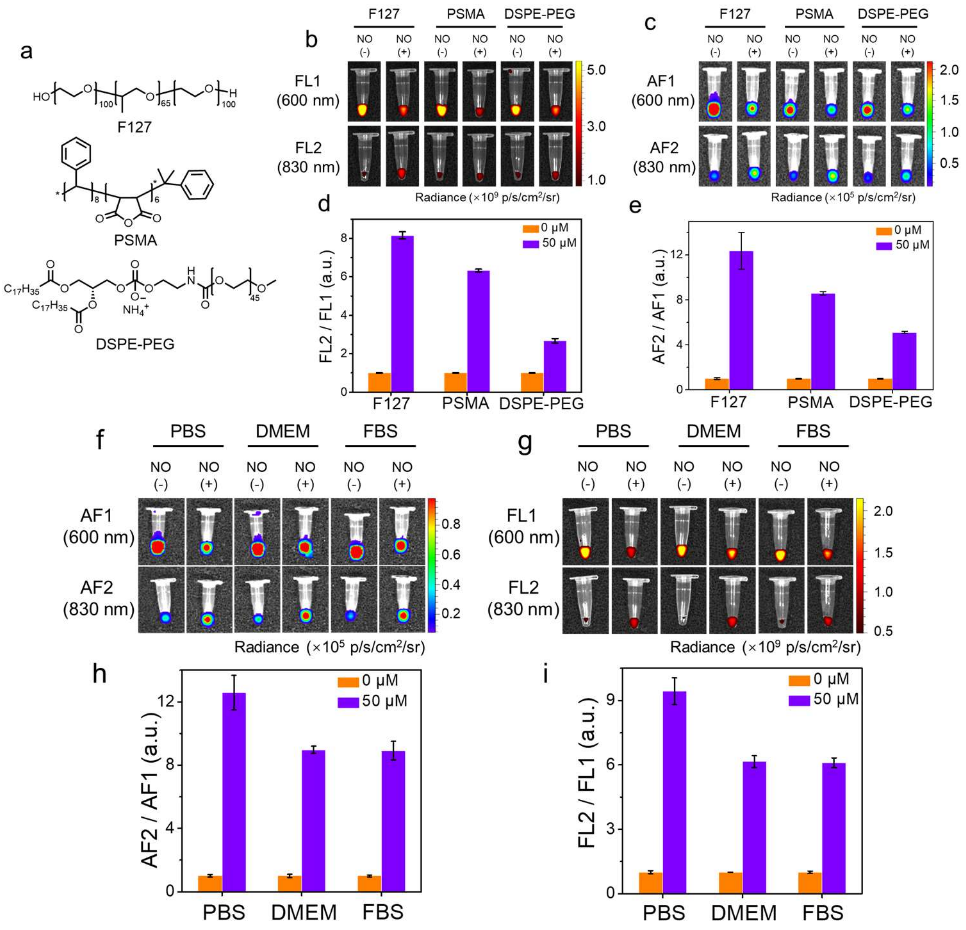

Supplementary Fig. 7 a, Chemical structures of the surfactants (F127, PSMA, and DSPE-PEG) used for the synthesis of RAN1-F, RAN1-P, and RAN1-D, respectively. **b**, Fluorescent and **c**, afterglow images of these surfactants-doped RAN1 (20 μg/mL) treated with different concentrations of NO (0 μM and 50 μM). **d**, **e**, Normalized fluorescence intensity ratios (FL2/FL1) and afterglow intensity ratios (AF2/AF1) in (**b**) and (**c**), respectively. **f**, Afterglow luminescent and **g**, fluorescent images of RAN1 (20 μg/mL) in PBS (pH = 5.4), DMEM medium, and FBS medium. **h**, **i**, Normalized afterglow intensity ratios (AF2/AF1) and fluorescence intensity ratios (FL2/FL1) in (**f**) and (**g**), respectively. Data are presented as mean values ± s.d. (n = 3). These results showed that F127-encapsulated nanoprobe resulted in the highest ratio of afterglow intensity (AF2/AF1) in the presence of NO and that no obvious interference from various buffer systems (e.g., PBS, DMEM, and FBS) prevented the detection of NO.

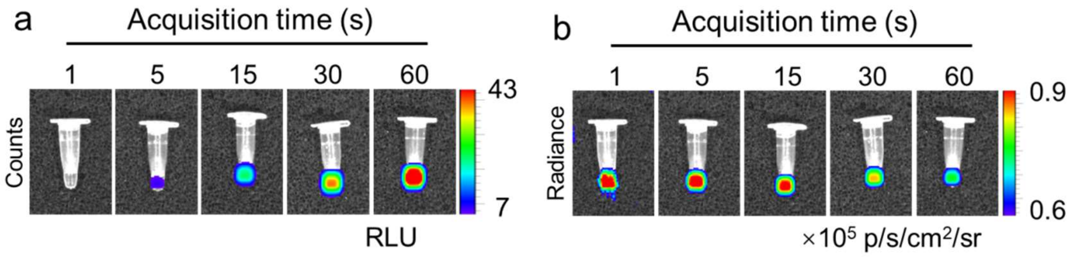

Supplementary Fig. 8 Afterglow luminescent images of RAN1 (20 μg/mL) expressed in units of **a**, counts (RLU) or **b**, radiance (p/s/cm^2/sr) with different acquisition times (0.1, 1.0, 5.0, 15 and 30 s). When radiance was selected as the acquisition mode, these results show that the afterglow images could be acquired as short as 1 s.

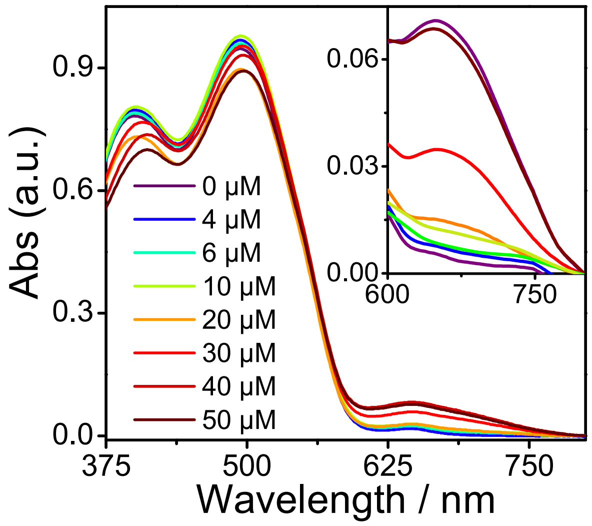

Supplementary Fig. 9 Absorption emission spectra of RAN1 (10 μg/mL) treated with different concentrations of NO solution (0-50 μM).

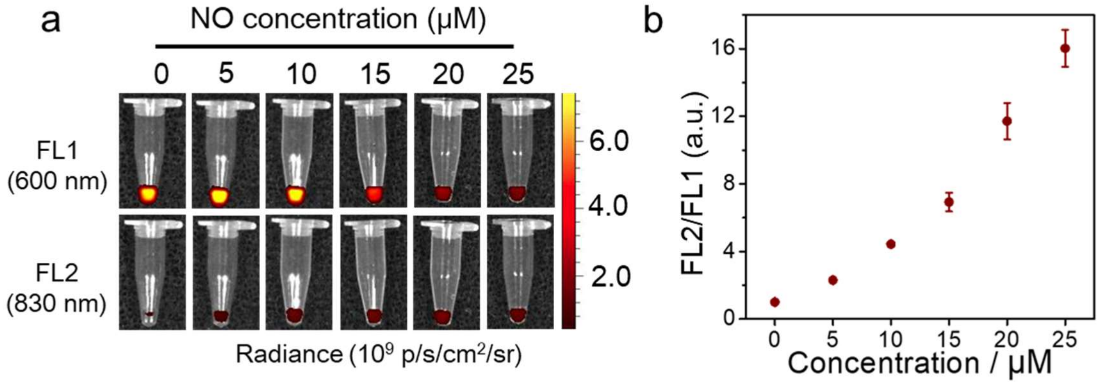

Supplementary Fig. 10 a, Fluorescent images of BDP-doped RAN1 (20 μg/mL) treated with different concentrations of NO. **b**, Normalized fluorescence intensity ratios (FL2/FL1) as a function of NO concentration. Data are presented as mean values ± s.d. (n = 3).

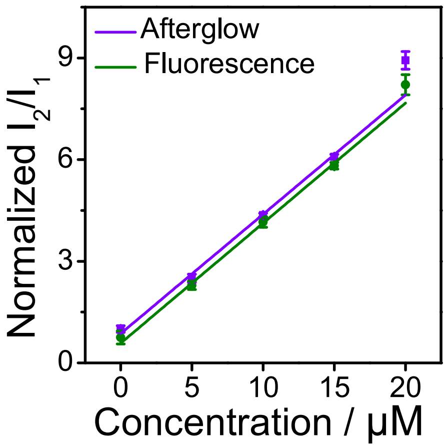

Supplementary Fig. 11 Plot of AF2/AF1 ratio for afterglow and FL2/FL1 ratio for fluorescence, respectively, with increasing NO concentration. Data are presented as mean values ± s.d. (n = 3).

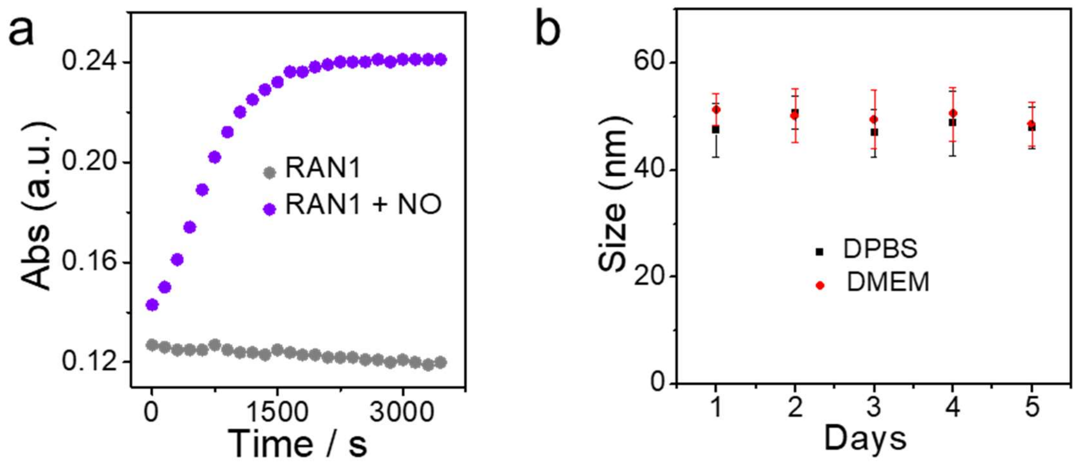

Supplementary Fig. 12 a, Dynamic absorption at 660 nm for RAN1 (10 μg/mL) responsive to NO (50 μM), or not. **b**, Hydrodynamic diameter of RAN1 in DPBS, and cell culture medium during 5 days. Data are presented as mean values ± s.d. (n = 3).

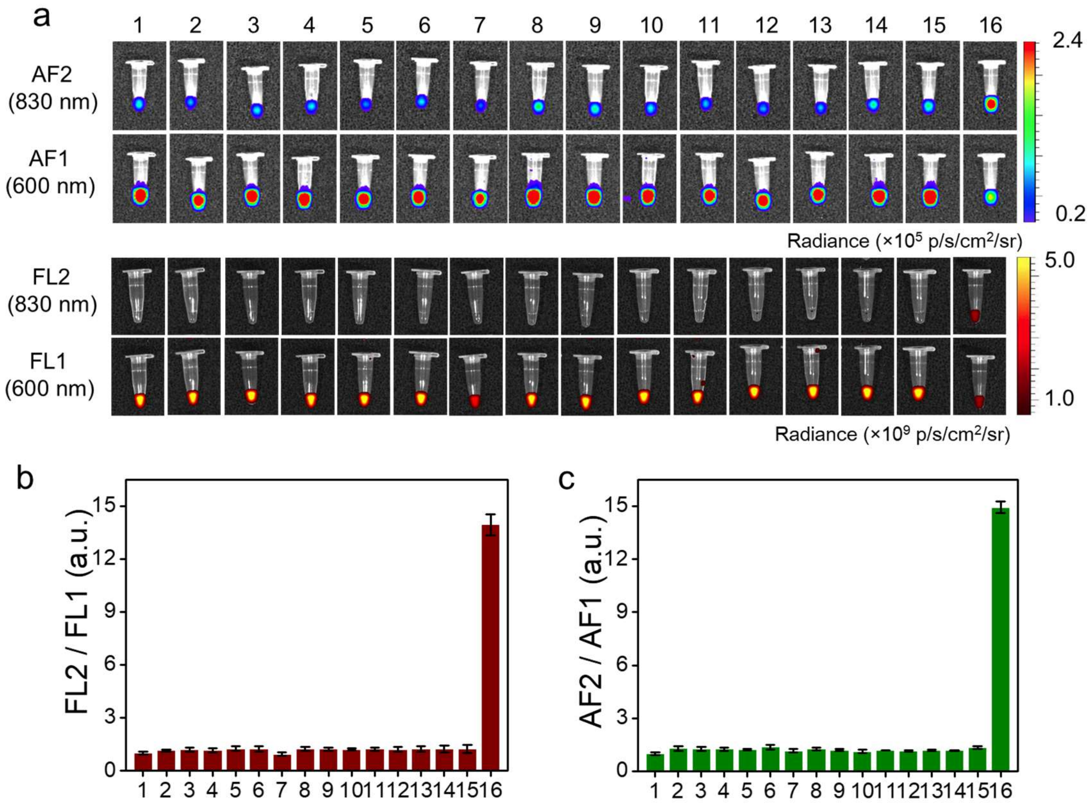

Supplementary Fig. 13 **a**, Afterglow and fluorescent images of RAN1 (20 μg/mL) upon incubation with different interference species. Normalized **b**, fluorescence and **c,** afterglow intensity ratios (FL2/FL1) in (**a**). 1. Blank, 2. Na^+ (10 mM), 3. K^+ (10 mM), 4. Ca^{2+} (10 mM), 5. Fe^{2+} (1 mM), 6. H_2O_2 (250 μM), 7. HClO (50 μM), 8. $ONOO^-$ (50 μM), 9. O_2^- (100 μM), 10. t-BuOOH (100 μM), 11. ·OH (100 μM), 12. Cys (500 μM), 13. GSH (1 mM), 14. H_2S (100 μM), 15. SO_3^{2-} (50 μM), and 16. NO (50 μM). Data are presented as mean values ± s.d. (n = 3).

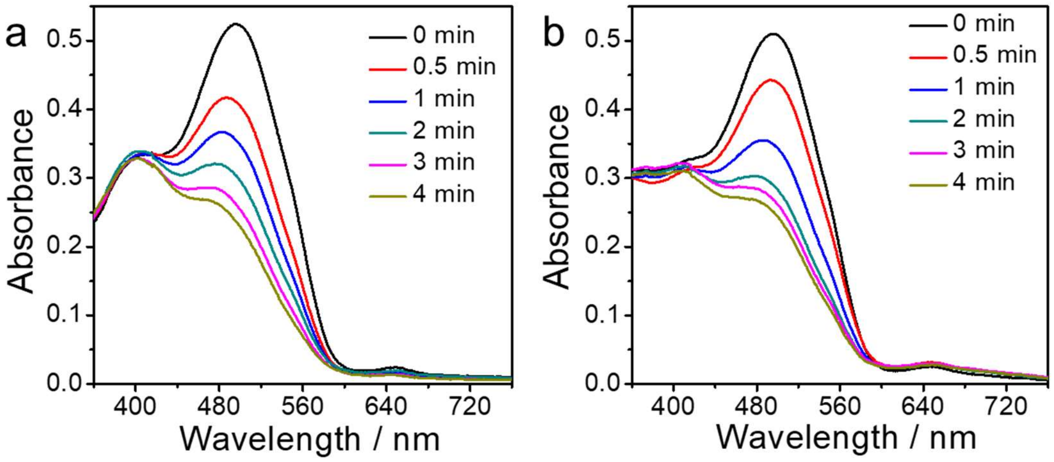

Supplementary Fig. 14 Absorption spectra of RAN1 (10 μg/mL) under the irradiation of 660 nm laser for different times **a**, with or **b**, without NRM doping.

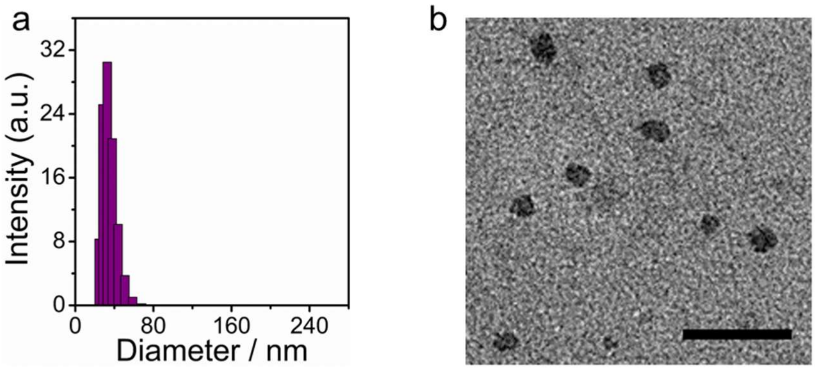

Supplementary Fig. 15 a, Dynamic light scattering (DLS) and **b**, transmission electron microscopy (TEM) image of RAN2. Scale bar: 100 nm. Each experiment was repeated for three times.

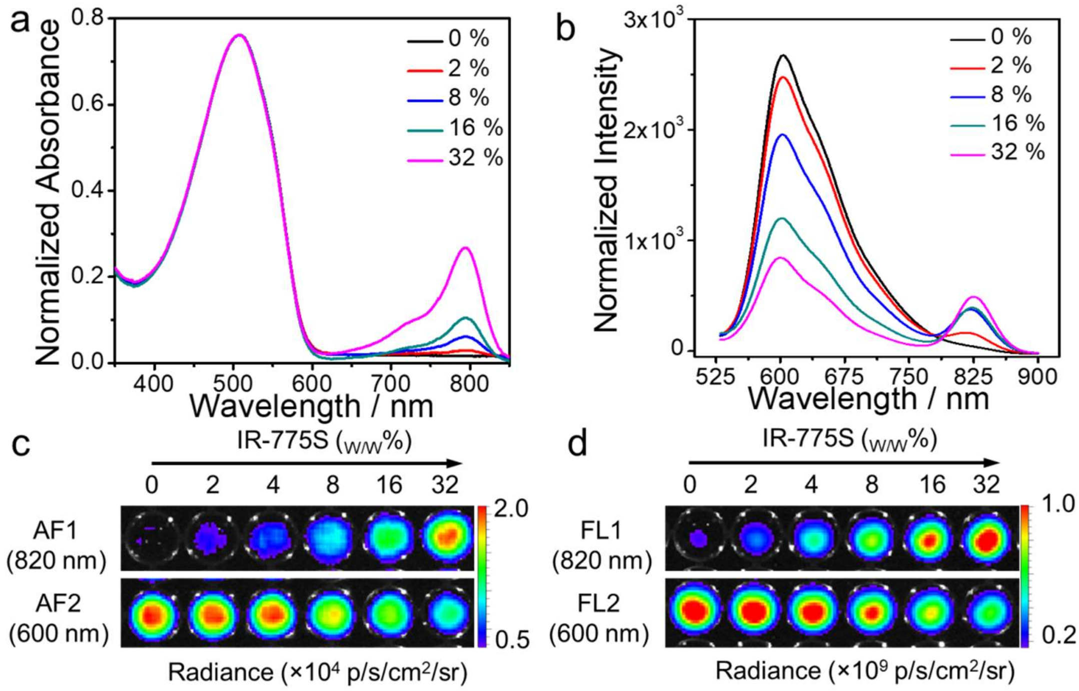

Supplementary Fig. 16 a, Normalized absorption spectra and **b**, fluorescence spectra of RAN2 (without photosensitizer) with different doping amounts of ORM. **c**, Afterglow luminescent images and **d,** fluorescent images of RAN2 with different doping amounts of ORM.

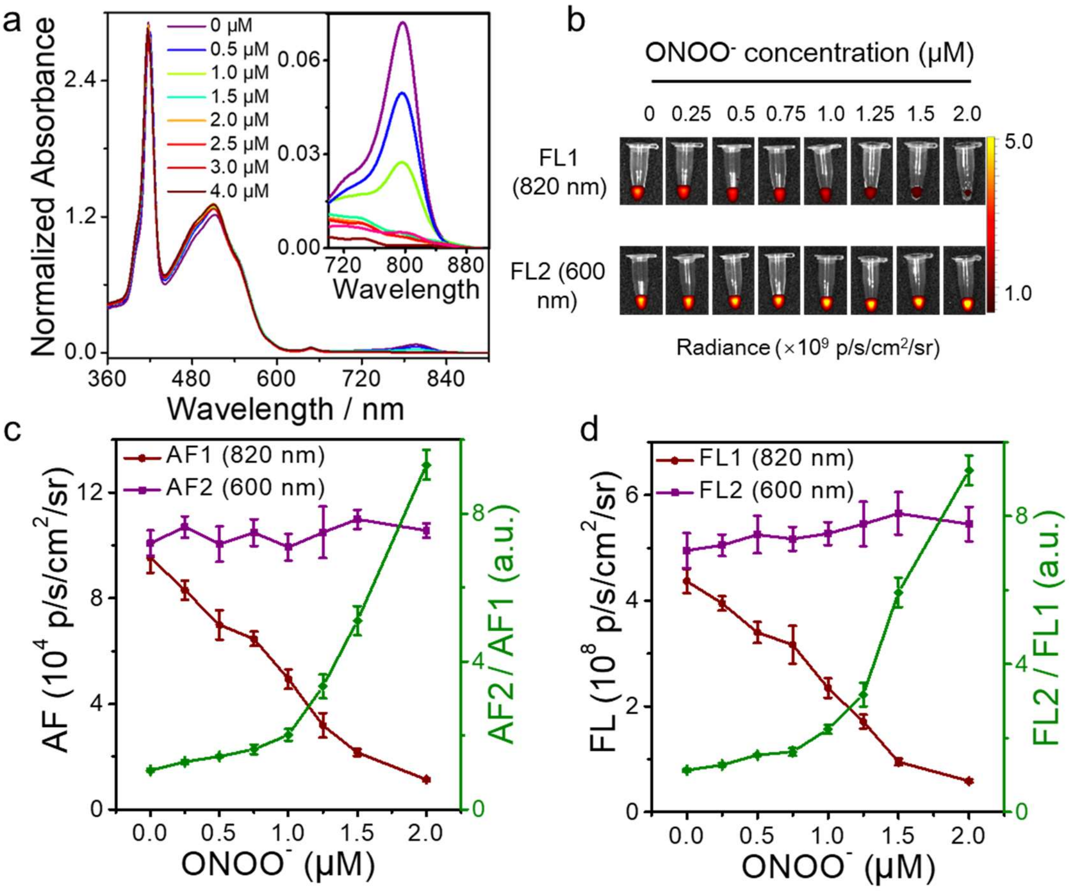

Supplementary Fig. 17 a, Normalized absorption spectra of RAN2 (2 μg/mL) incubated with different concentrations of $ONOO^-$. **b**, Afterglow luminescent images and fluorescent images of doped RAN2 (2 μg/mL) treated with different concentrations of $ONOO^-$. **c**, Quantification of afterglow luminescence intensities (AF1 and AF2) and normalized intensity ratios (AF2/AF1, green line) as a function of $ONOO^-$. **d**, Quantification of the fluorescence intensities (FL1 and FL2) and normalized intensity ratios (FL2/FL1, green line) as a function of $ONOO^-$ concentration. Data are presented as mean values ± s.d. (n = 3).

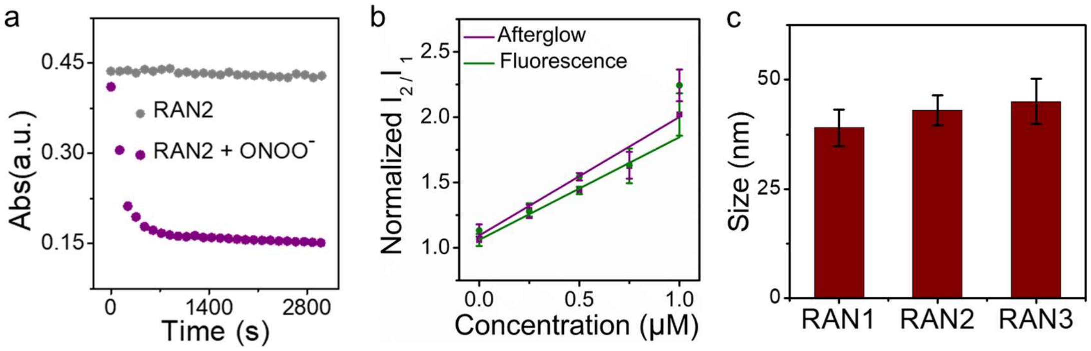

Supplementary Fig. 18 a, Dynamic absorption at 780 nm for RAN2 (2 μg/mL) responsive to $ONOO^-$ (4.0 μM), or not. **b**, Plot of the normalized luminescence intensity ratios (AF2/AF1 and FL2/FL1) of RAN2 (2 μg/mL) with the concentration of $ONOO^-$ from 0 to 1.0 μM. **c,** The hydrodynamic diameter of RAN1, RAN2 and RAN3 in PBS buffer solutions. Data are presented as mean values ± s.d. (n = 3).

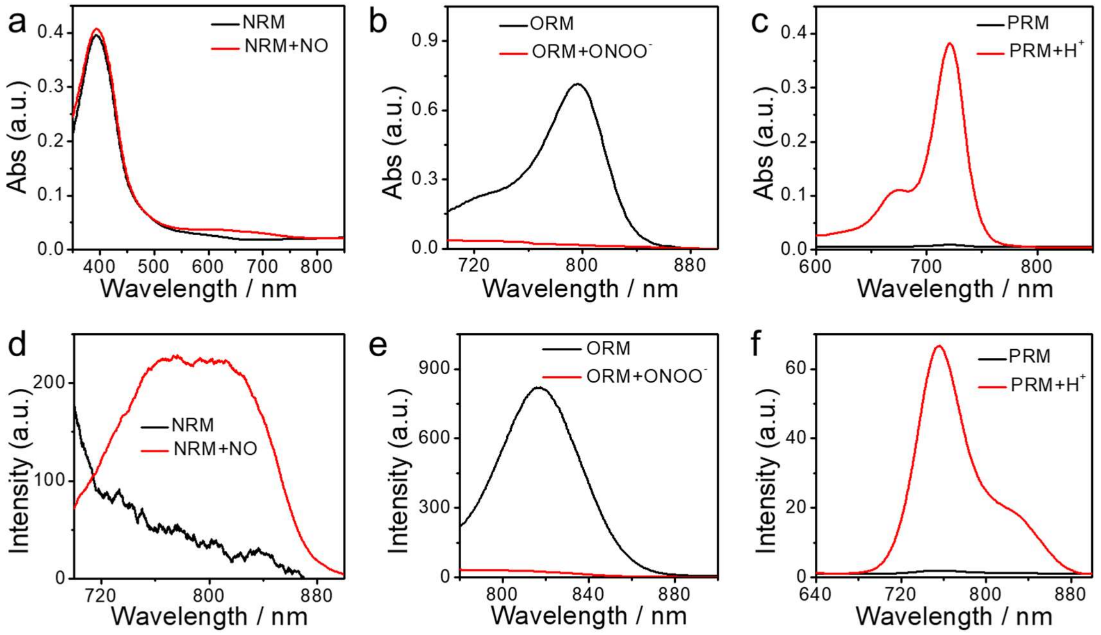

Supplementary Fig. 19 a-c, d-f, Absorption (**a-c**) and fluorescence spectra (**d-f**) of 5 μM NRM, ORM, and PRM in the absence or presence of their respective targets (25 μM NO, 4.0 μM $ONOO^-$ and pH 3.0, respectively) in PBS solutions. The fluorescence excitation was set at 660 nm, 760 nm, and 710 nm, respectively.

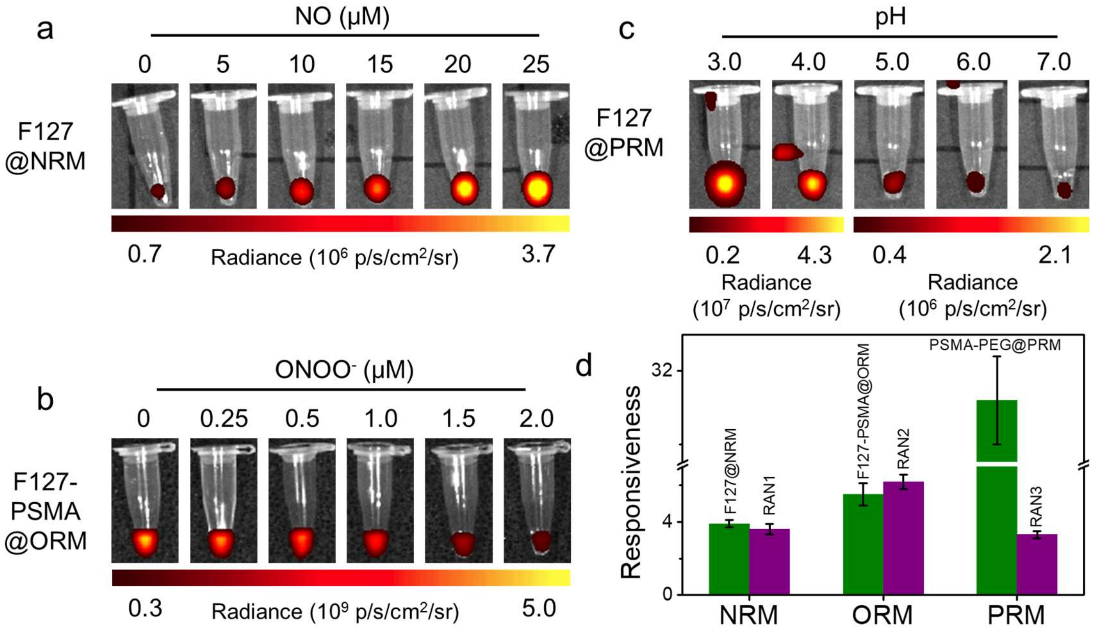

Supplementary Fig. 20 Fluorescence images of **a,** F127@NRM, **b,** F127-PSMA@ORM, and **c,** F127@PRM in NO, $ONOO^-$ buffer solutions with different concentrations and PBS with different pH values, respectively. **d,** The responsiveness difference between of ratiometric nanoplatform and responsive molecules (NRM, ORM or PRM) through comparing the signal enhancement at maximum fluorescence emission channel. Data are presented as mean values ± s.d. (n = 3).

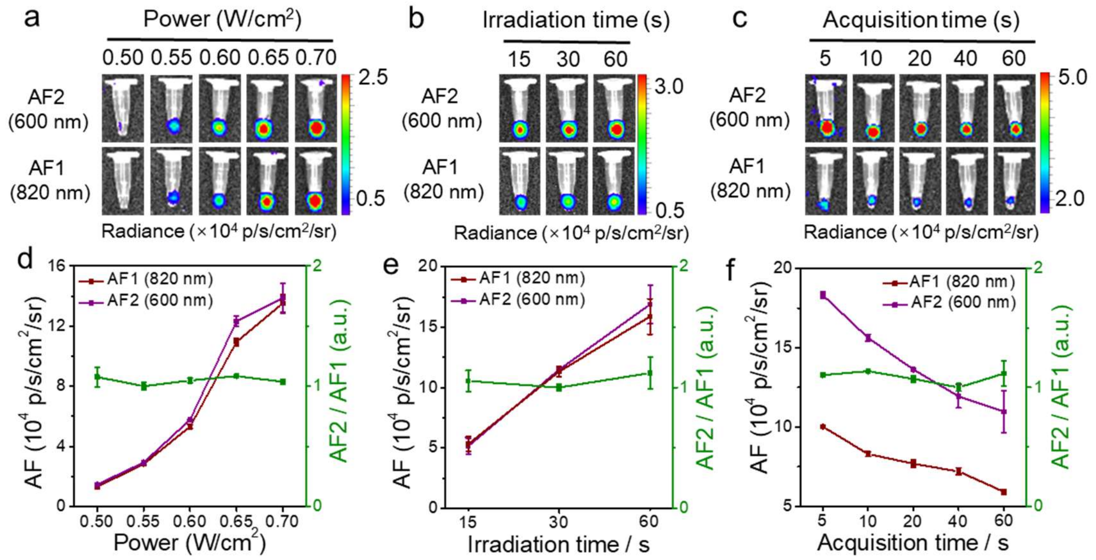

Supplementary Fig. 21 Representative afterglow luminescent images of RAN2 (2 μg/mL) as a function of **a**, laser power **b**, irradiation time and **c**, acquisition time. Quantification of the afterglow luminescence intensities (AF1 and AF2) and normalized intensity ratios (AF2/AF1, green line) as a function of **d**, laser power **e**, irradiation time and **f**, acquisition time. Data are presented as mean values ± s.d. (n = 3).

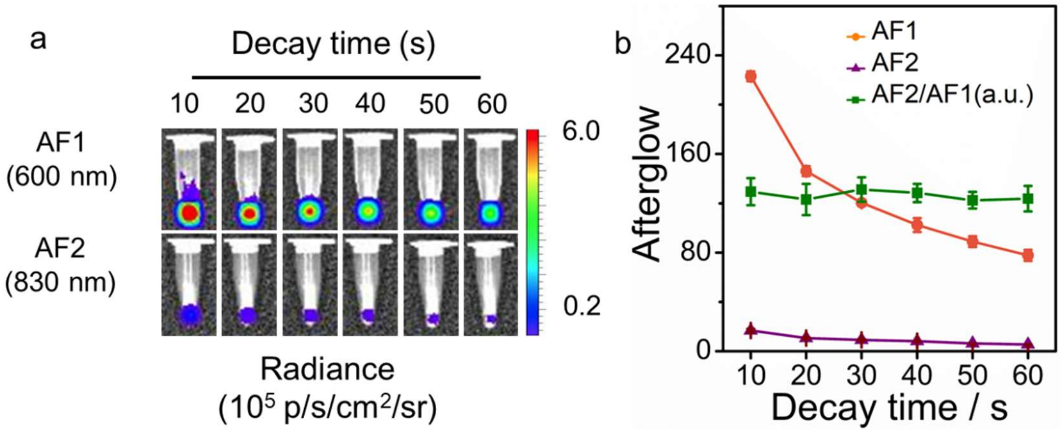

Supplementary Fig. 22 a, Representative afterglow images of RAN1 (20 μg/mL) as a function of decayed time in the absence of NO. **b**, The afterglow luminescence intensity of AF1 and AF2 and normalized intensity ratio (AF2/AF1) as a function of decayed time in RAN1 (20 μg/mL) in (**a**). Data are presented as mean values ± s.d. (n = 3).

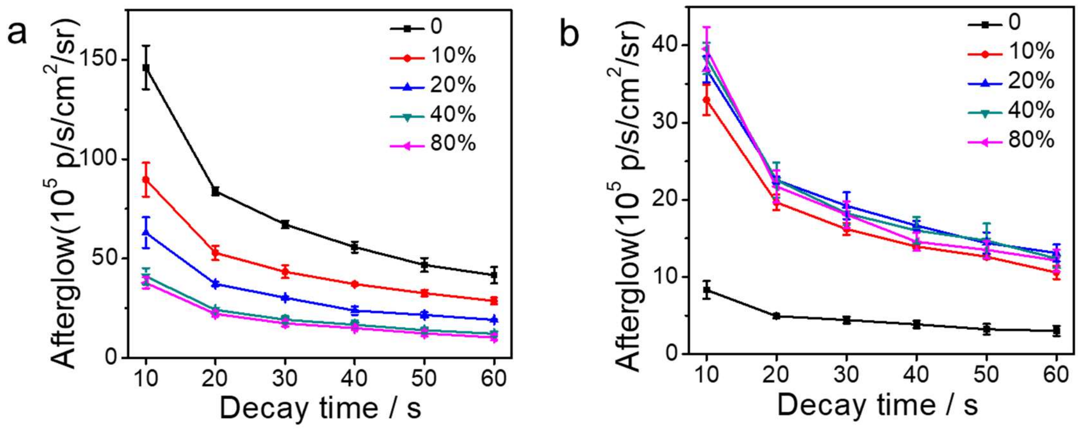

Supplementary Fig. 23 The afterglow luminescence intensity of **a**, AF1 and **b**, AF2 as a function of decayed time in RAN1 (20 μg/mL) with different doping amount of NRM in the presence of NO (50 μM). Data are presented as mean values ± s.d. (n = 3).

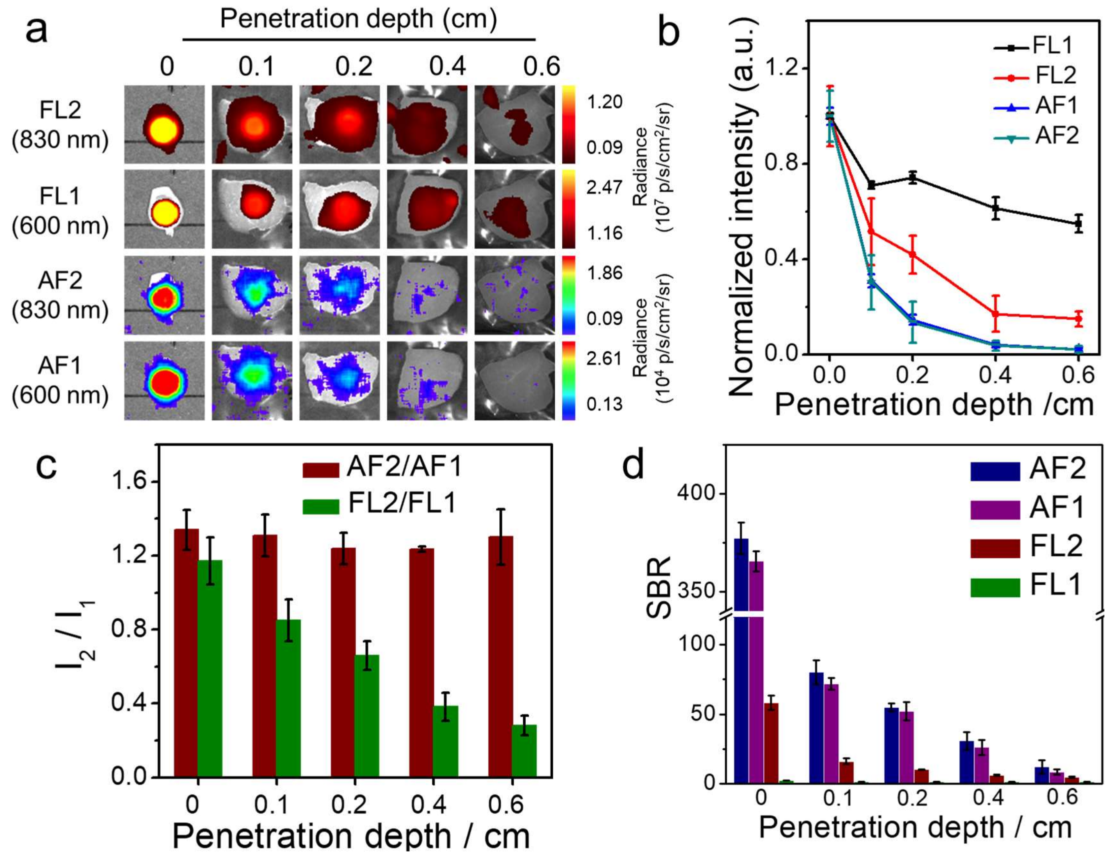

Supplementary Fig. 24 a, Fluorescent and afterglow images of RAN1 (20 μg/mL) through chicken tissues of different thickness in the presence of NO (50 μM). **b**, Normalized fluorescence intensities (FL1 and FL2) and afterglow luminescence intensities (AF1 and AF2) as a function of penetration depth in (**a**). The signal intensity of chicken tissue with the thickness at 0 cm was defined as one unit. **c**, The corresponding fluorescence ratio (FL2/FL1) and afterglow ratio (AF2/AF1) as a function of penetration depth. **d**, SBR for FL1, FL2, AF1, and AF2 as function of penetration depth in (**a**), respectively. Data are presented as mean values ± s.d. (n = 3).

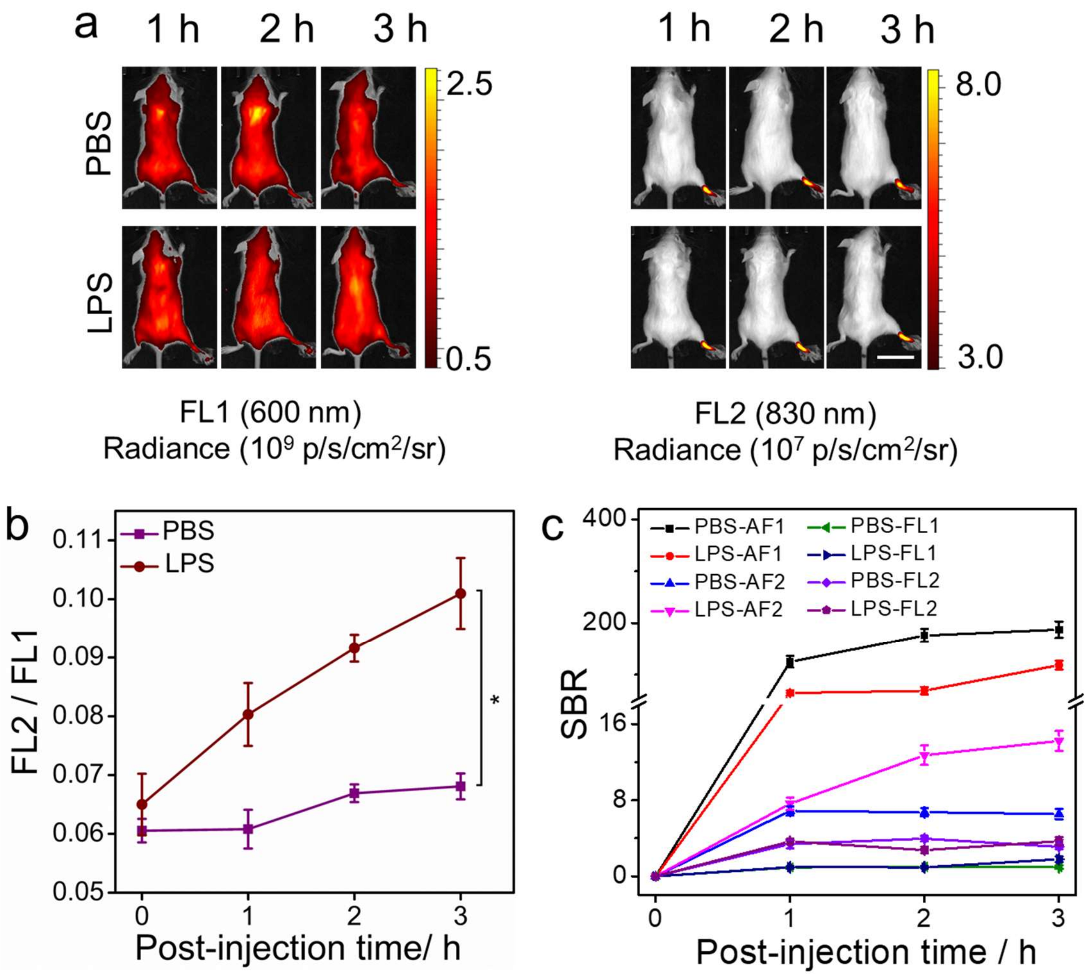

Supplementary Fig. 25 a, Representative fluorescent images as a function of post-injection time of RAN1 (10 μg/mL) in LPS- or PBS-pretreated mice. Scale bar: 2 cm. **b**, The corresponding fluorescence intensity ratios from (**a**). **c**, SBR for fluorescence or afterglow. Data are presented as mean values ± s.d. (n = 3).

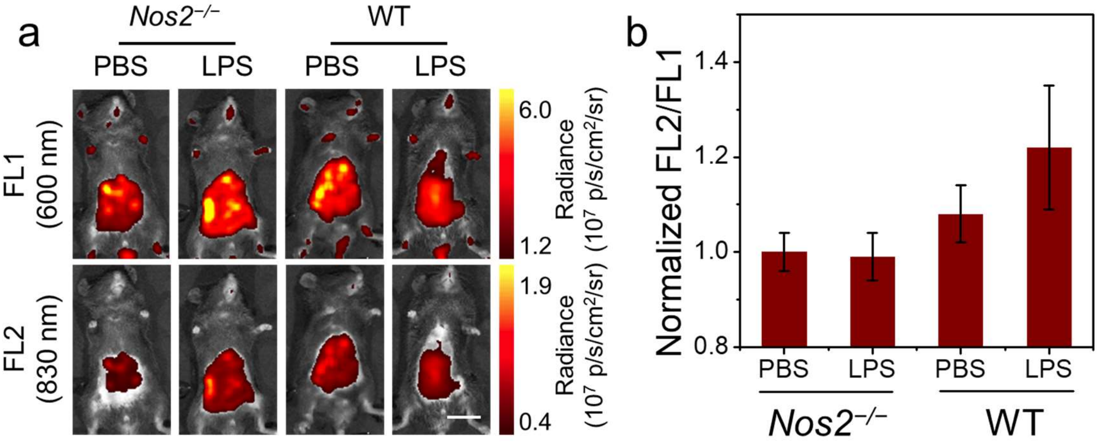

Supplementary Fig. 26 a, Representative fluorescent images of *Nos2*$^{-/-}$ and WT mice upon post-intravenous injection of RAN1 (200 μg/mL) in LPS-induced liver injury model. Scale bar: 2 cm. **b,** Normalized afterglow intensity ratio in (**a**). Data are presented as mean values ± s.d. (n = 3).

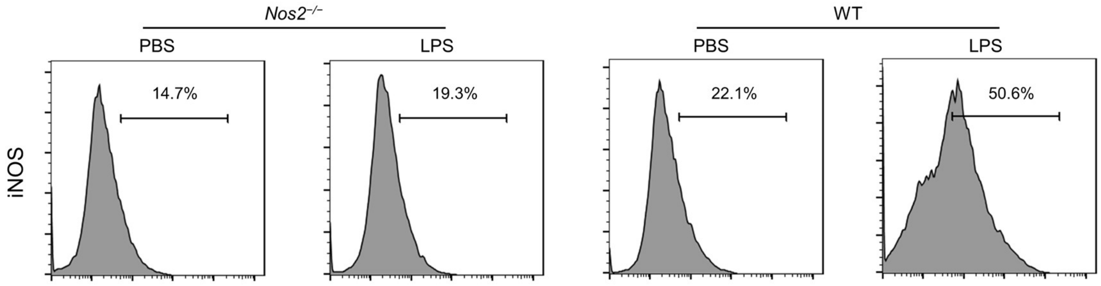

Supplementary Fig. 27 Flow cytometric analysis showed the expression of iNOS in liver of *Nos2*$^{-/-}$ and WT mice upon different administrations. Each experiment was repeated for three times.

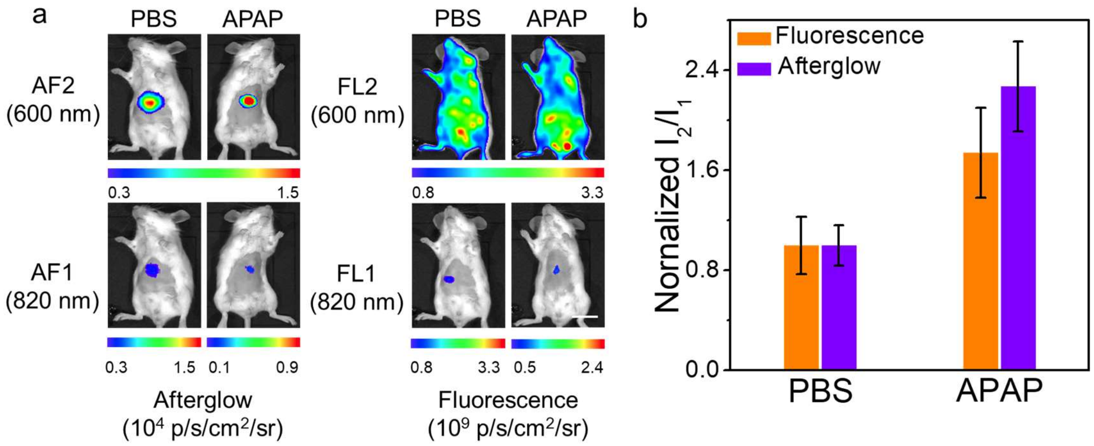

Supplementary Fig. 28 **a**, Representative images of BALB/c mice which received PBS and APAP (200 mg/kg, 100 μL, intraperitoneally) for 1 h, followed by RAN2 (100 μL, 40 μg/mL, intravenously) for different durations, respectively. Scale bar: 2 cm. **b**, Normalized intensity ratios (AF2/AF1 and FL2/FL1) in (**a**). Data are presented as mean values ± s.d. (n = 3).

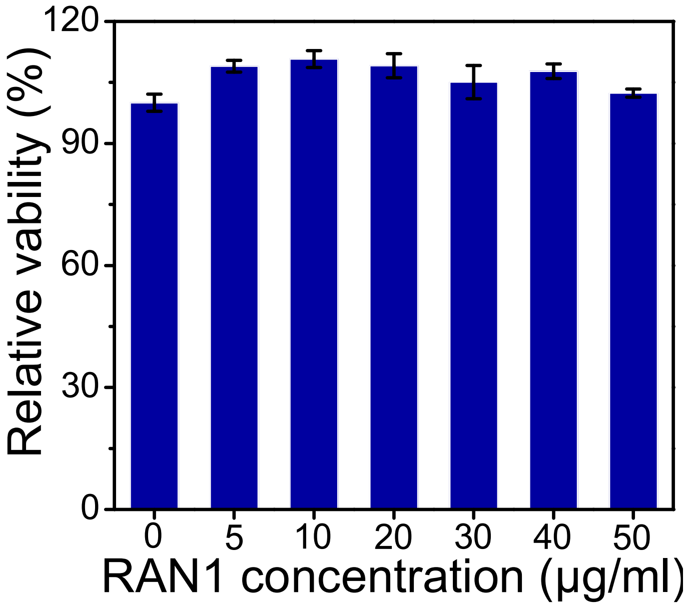

Supplementary Fig. 29 Relative viabilities of 4T1 cancer cells incubated with RAN1 with different concentrations for 24 h, as measured by MTT assay. Data are presented as mean values ± s.d. (n = 3).

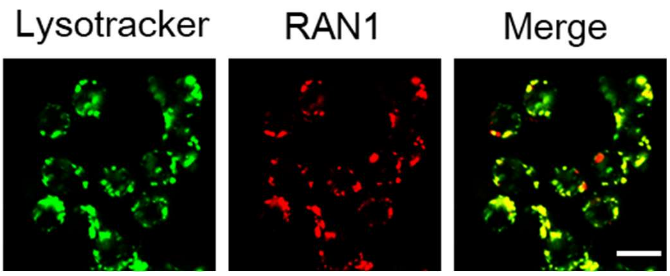

Supplementary Fig. 30 Colocalization experiments in RAW264.7 cells. Confocal fluorescent image of cells incubated with RAN1 (10 μg/mL) for 4 h and then LysoTracker Green (100 nM) for 30 min. Green channel: λ_{ex} = 488 nm, λ_{em} = 500-560 nm; Red channel: λ_{ex}= 559 nm, λ_{em}= 580-650 nm. Scale bar: 20 μm. Each experiment was repeated for three times.

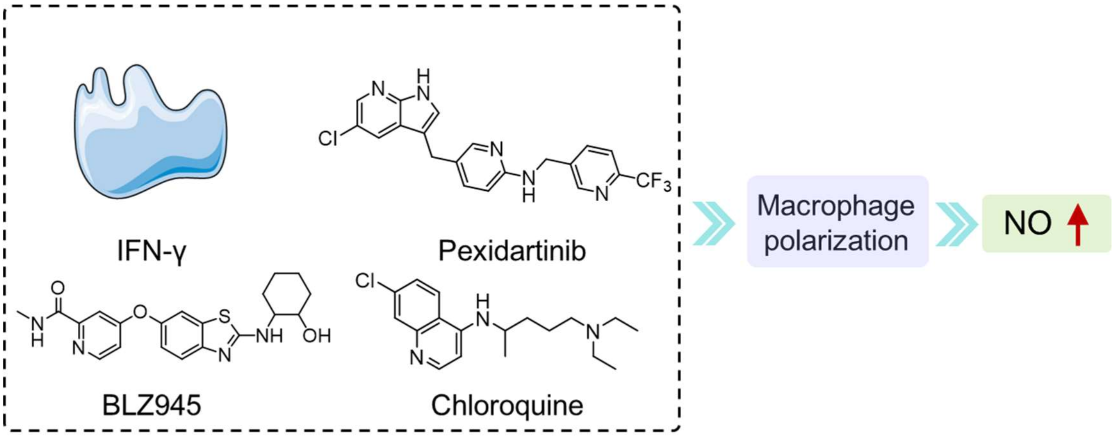

Supplementary Fig. 31 Chemical structures of different modulators (IFN-γ, BLZ945, pexidartinib, chloroquine) for activation of immune responses.

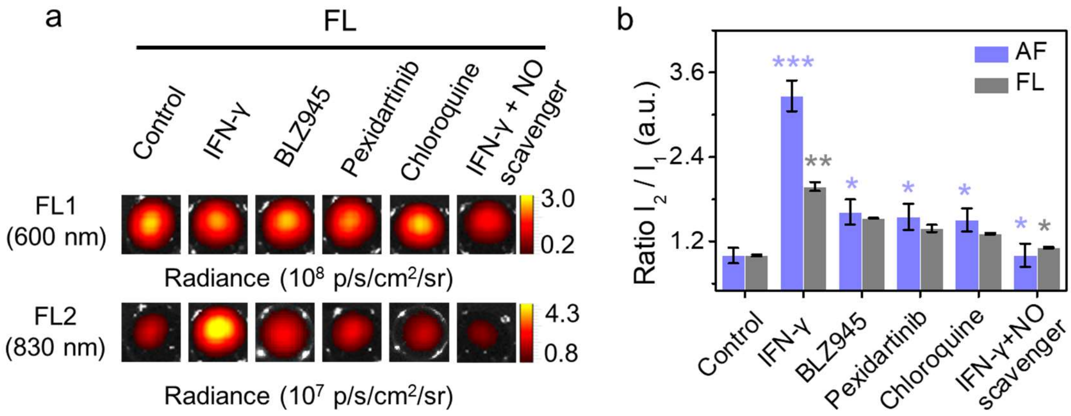

Supplementary Fig. 32 a, Representative fluorescent images of RAW 264.7 macrophages treated with different modulators and then with RAN1 (10 μg/mL). **b,** Normalized AF2/AF1 ratio for afterglow and FL2/FL1 ratio for fluorescence. Data are presented as mean values ± s.d. (****p* < 0.001, ***p* < 0.01, *p < 0.05, n = 3). Statistical differences were analyzed by Student's t test.

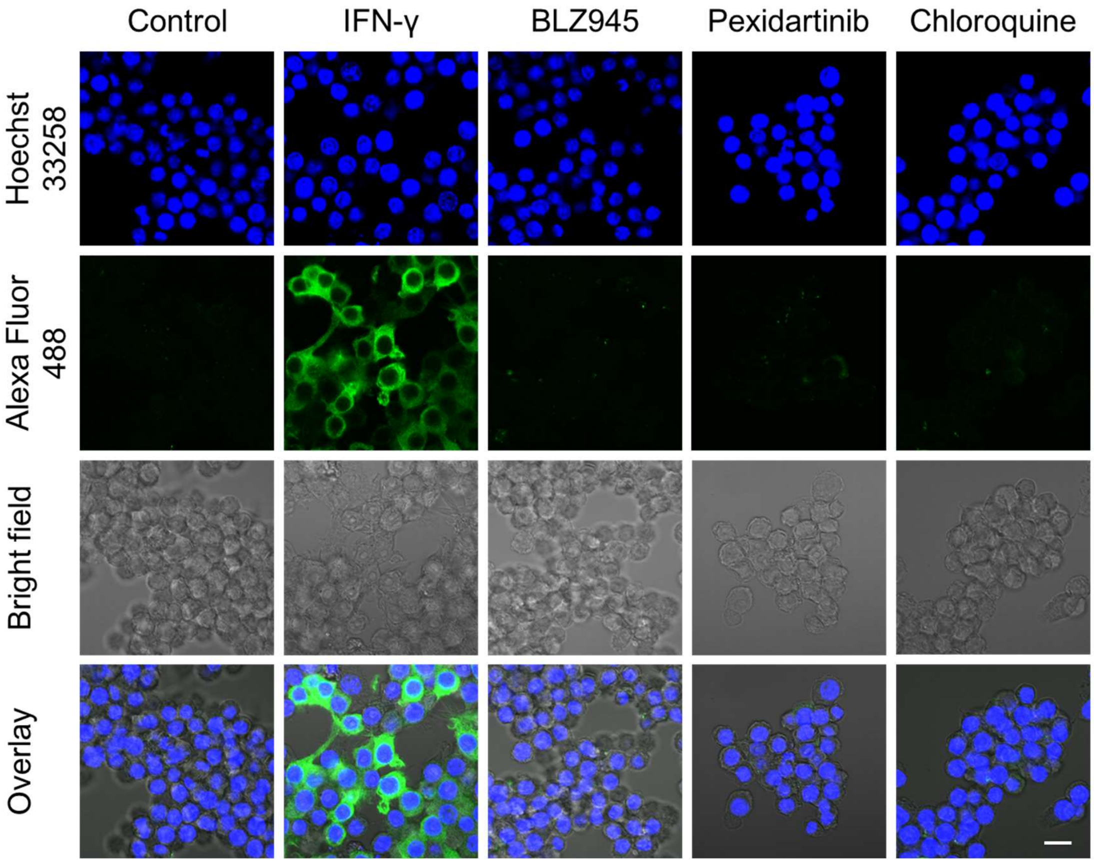

Supplementary Fig. 33 Immunofluorescence staining of RAW264.7 cells incubated with different polarization modulators. Green signals indicate iNOS stained with Anti-iNOS antibody and blue signals represent the cell nucleus (Hoechst 33258). Blue channel: λ_{ex} = 405 nm, λ_{em} = 425-475 nm; Green channel: λ_{ex}= 488 nm, λ_{em}= 500-560 nm. Scale bar: 20 μm. Each experiment was repeated for three times.

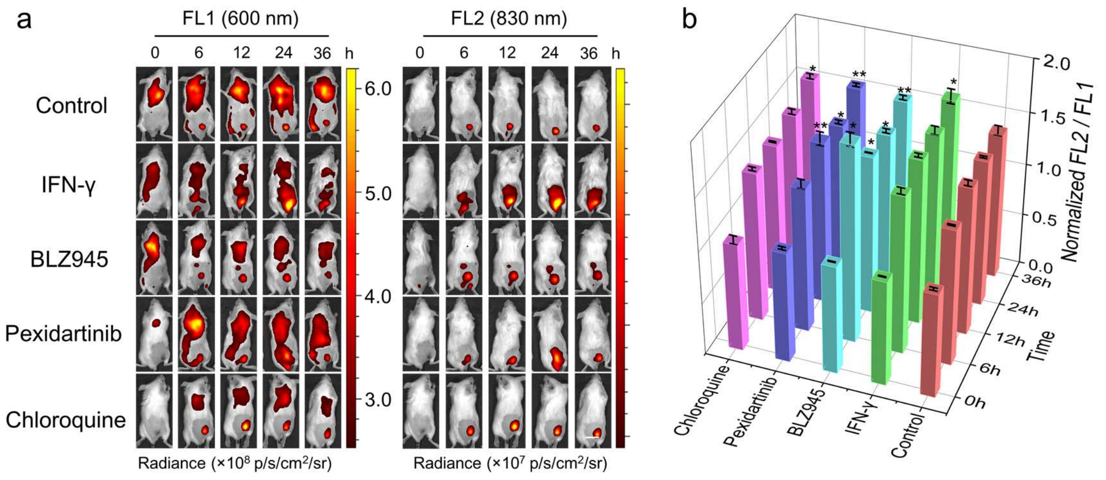

Supplementary Fig. 34 **a**, Representative fluorescent images of 4T1 tumor-bearing mice treated with modulators (i.t.) and then i.v.-injected with RAN1 (200 μg/mL). Scale bar: 2 cm. **b**, The corresponding quantification of fluorescence intensity ratios (FL2/FL1) in (**a**). Data are presented as mean values ± s.d. (***p* < 0.01, *p < 0.05, n = 3). Statistical differences were analyzed by Student's t test.

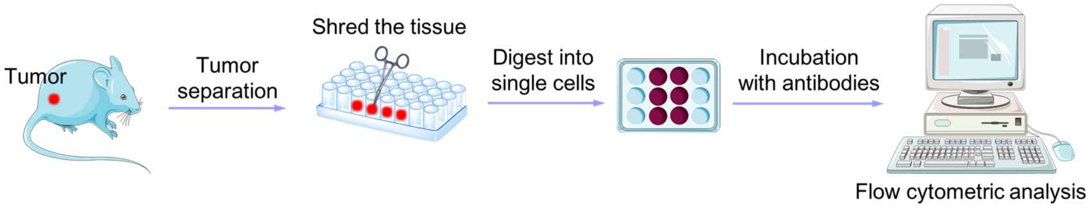

Supplementary Fig. 35 Schematic representation showing isolation of TAMs from tumor-bearing mice.

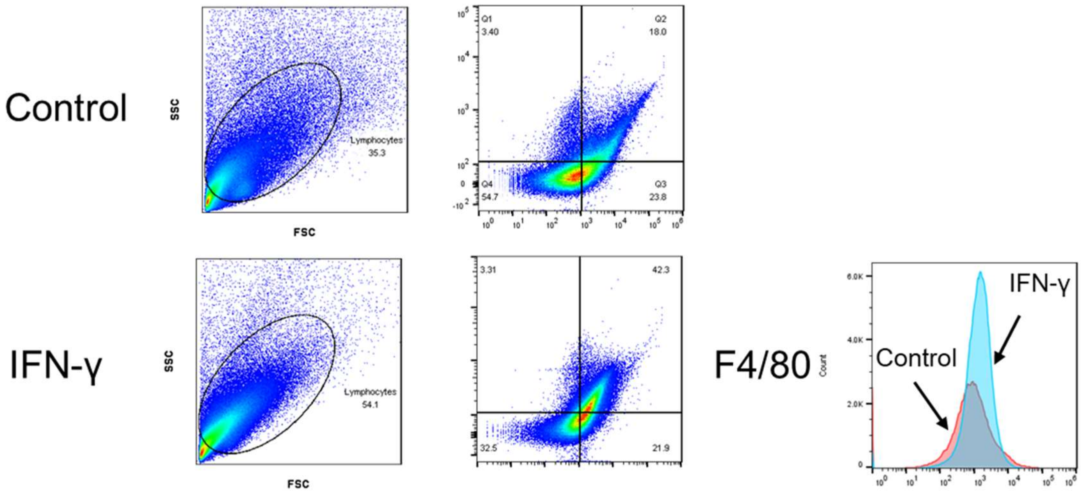

Supplementary Fig. 36 Flow cytometry gating strategy for TAM immune profiling.

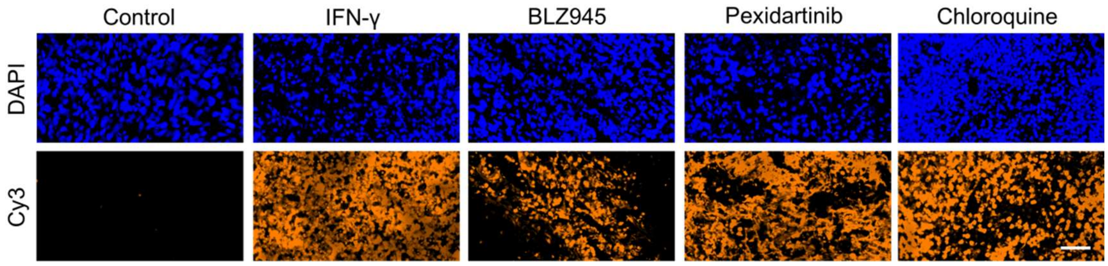

Supplementary Fig. 37 Representative TUNEL-stained tumor slices of each group on the second day post-injection. The sections stained with orange represented the apoptotic cells. Scale bar: 100 μm. Each experiment was repeated for three times.

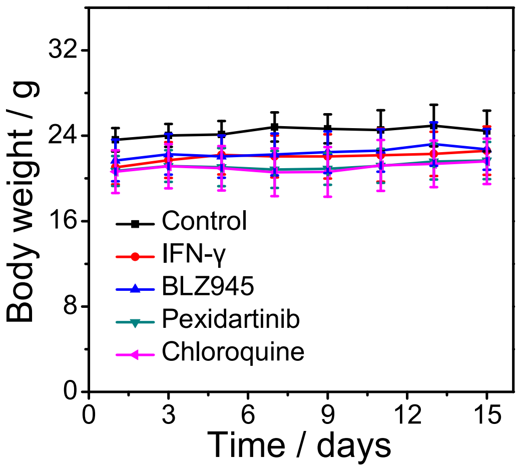

Supplementary Fig. 38 Changes in body weight of BALB/C mice in each group during macrophage-mediated immunotherapy. Data are presented as mean values ± s.d. (n = 3).

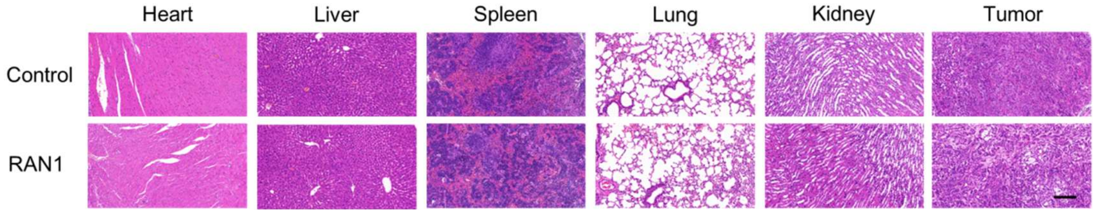

Supplementary Fig. 39 Representative H&E-stained major organs after systematic administration of RAN1 (20 μg/mL, 200 μL) or saline *via* i.v. injection. Scale bar: 100 μm. Each experiment was repeated for three times.

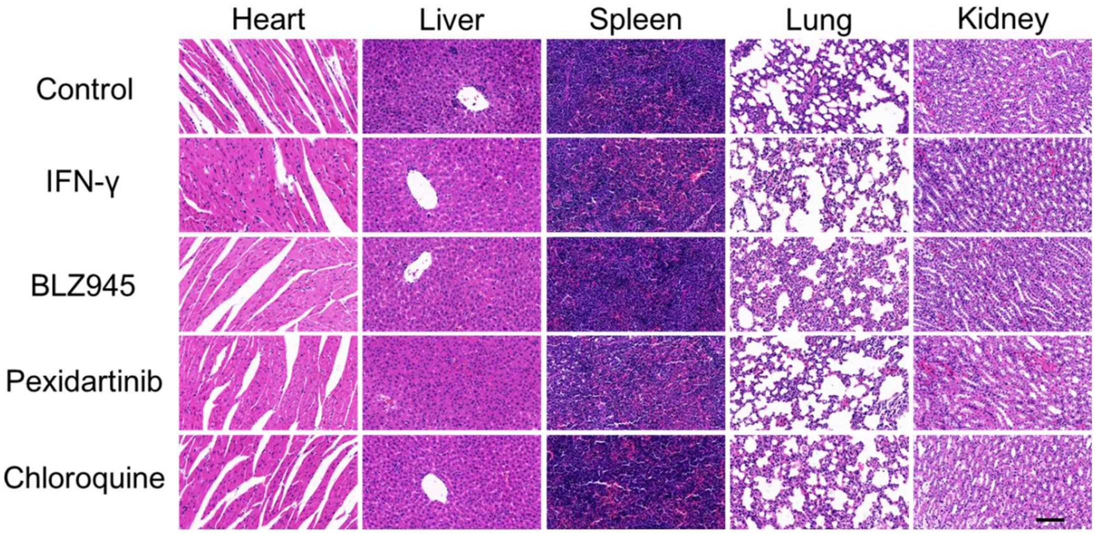

Supplementary Fig. 40 Representative H&E-stained major organs from different groups at 15 days post-administration. Scale bar: 100 μm. Each experiment was repeated for three times.

Supplementary Table S1 Explanation of the jargon in this manuscript.

Jargon	Explanation
**Afterglow**	An internal luminescence pathway that occurs after photo-excitation
**Afterglow substrate (MEHPPV)**	Materials capable of generating afterglow luminescence after photo-excitation
**Afterglow initiators (TPP or BDP)**	Materials capable of inducing afterglow substrates to emit luminescence after photo-excitation
**Responsive molecules (NRM, ORM, and PRM)**	Activatable molecular probes that indicate the changes of targets (NO, $ONOO^-$ and pH)
**Afterglow resonance energy transfer**	A resonance energy transfer process between the afterglow substrate (energy donor) and the acceptor fluorophore (responsive molecule)
**Ratiometric afterglow imaging**	An afterglow imaging mode in which the two afterglow outputs exhibit simultaneously changing signals (eg. “seesaw” type) upon interaction with the targets
**Macrophage polarization**	Macrophages enable to change their activation states in response to growth factors and external cues, and potentially any other entity capable of being recognized by macrophages
**Tumor microenvironment (TME)**	The internal environment in which tumor cells generate and survive, and is the location of tumor occurrence, growth and metastasis
**Tumor-associated macrophages (TAMs)**	Macrophages that infiltrate in tumor tissue and are also the most abundant immune cell in the tumor microenvironment.
**CD86, CD80, iNOS**	Markers of M1-like macrophage

Supplementary Note 1

MS and NMR Spectra

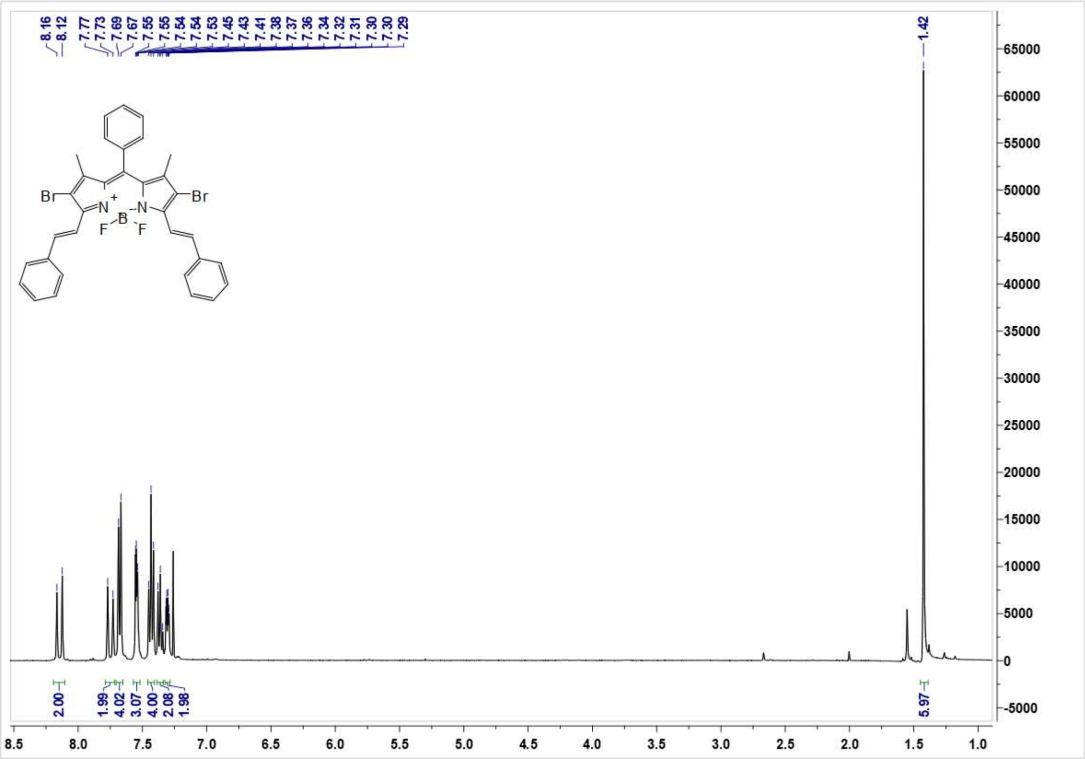

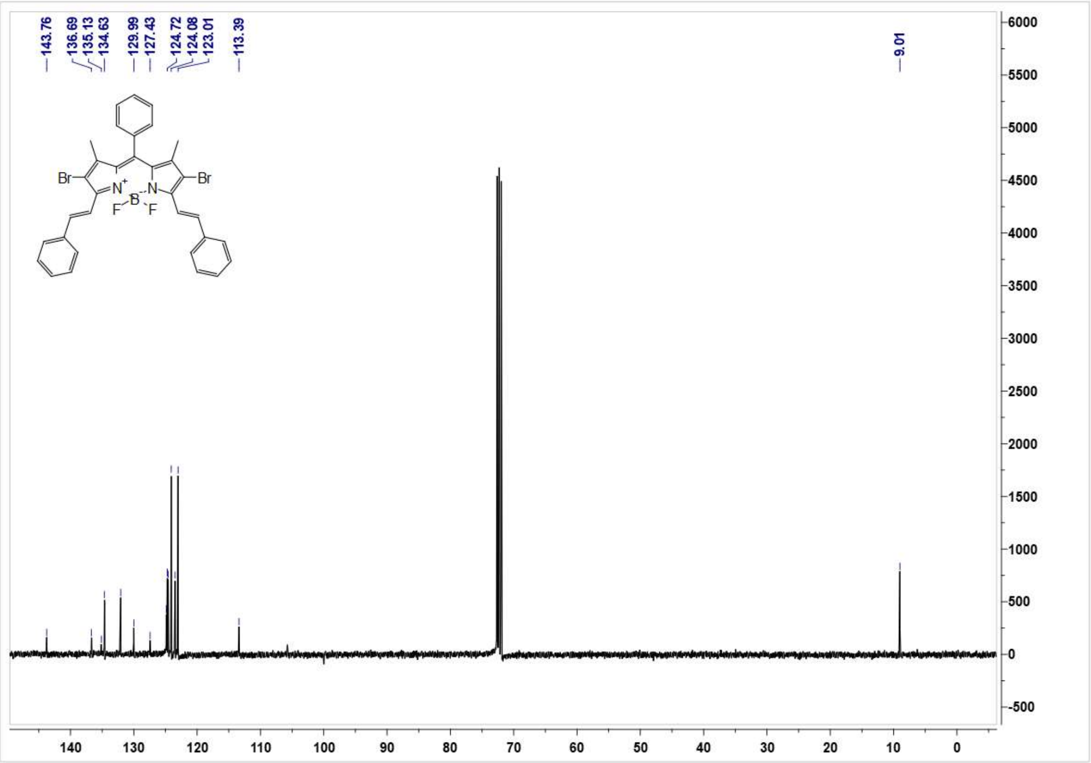
143.76
136.69
135.13
134.63
129.99
127.43
124.72
124.08
123.01
113.39
9.01
Br
Br
N
N
B
F
F
6000
5500
5000
4500
4000
3500
3000
2500
2000
1500
1000
500
0
-500
140
130
120
110
100
90
80
70
60
50
40
30
20
10
0

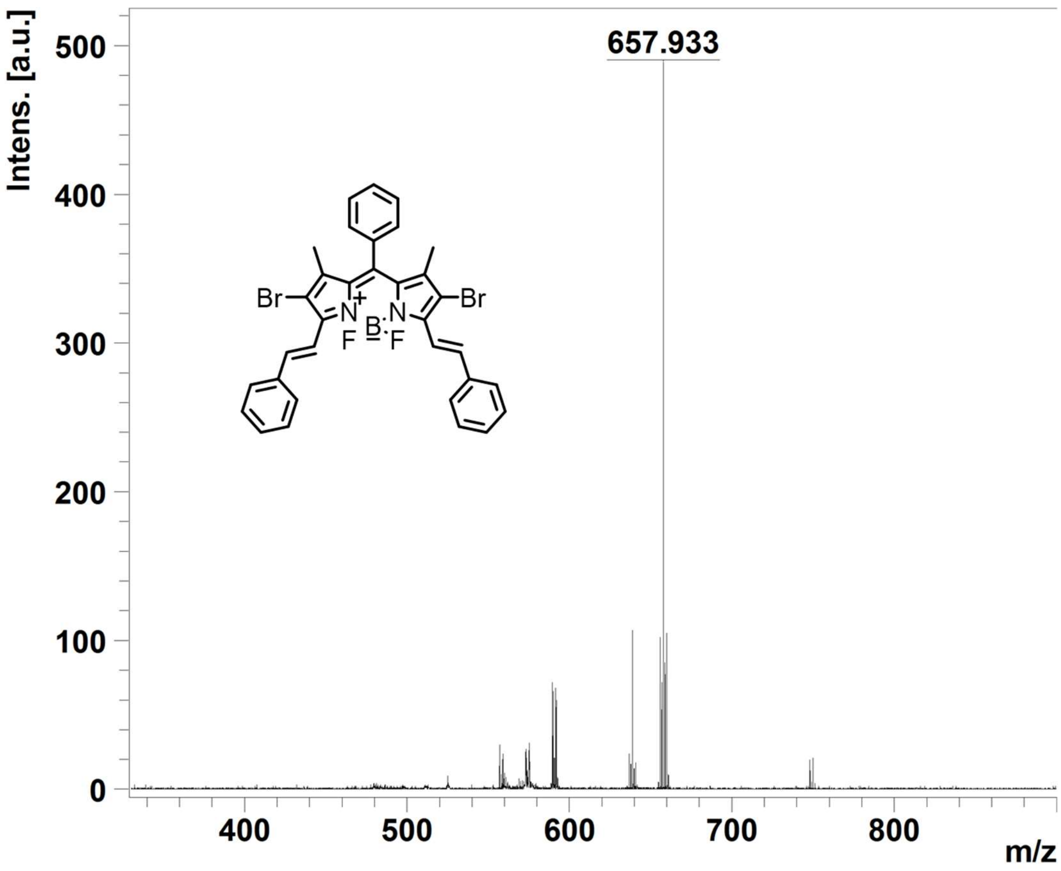
657.933
Intens. [a.u.]
500
400
300
200
100
0
400
500
600
700
800
m/z
Br
Br
N
N
B
F
F

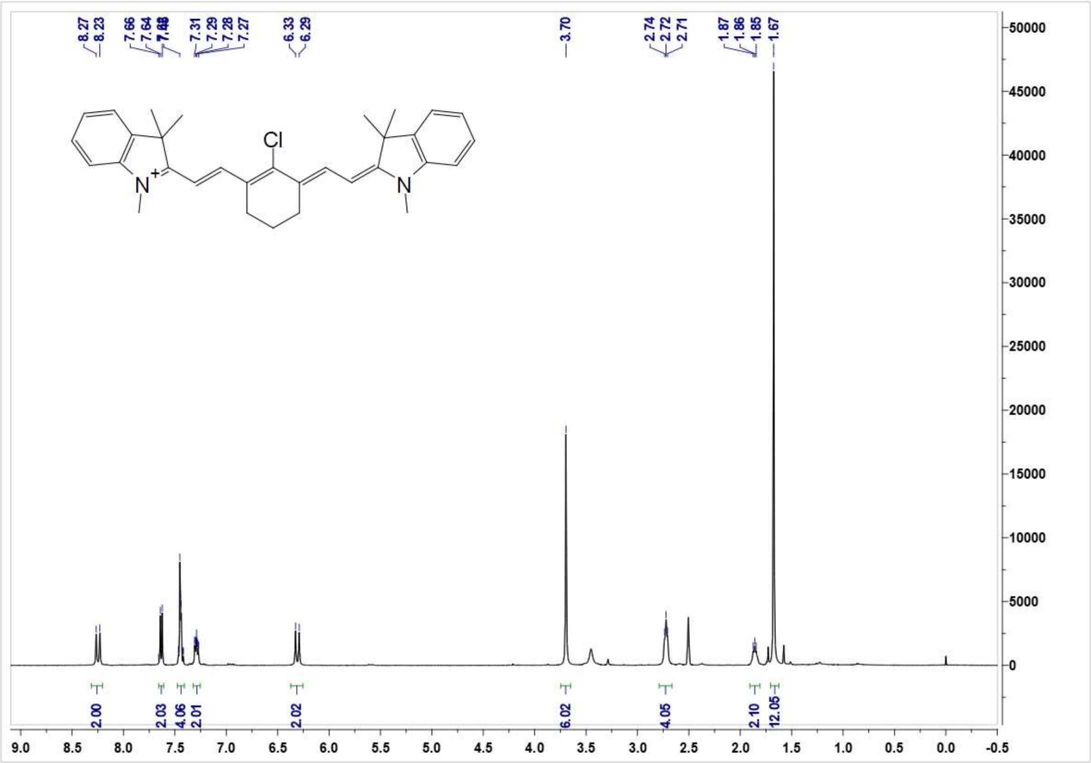
8.27
8.23
7.66
7.64
7.31
7.29
7.28
7.27
6.33
6.29
3.70
2.74
2.72
2.71
1.87
1.86
1.85
1.67
Cl
N+
N
2.00
2.03
4.06
2.01
2.02
6.02
4.05
2.10
12.05
50000
45000
40000
35000
30000
25000
20000
15000
10000
5000
0
9.0
8.5
8.0
7.5
7.0
6.5
6.0
5.5
5.0
4.5
4.0
3.5
3.0
2.5
2.0
1.5
1.0
0.5
0.0
-0.5

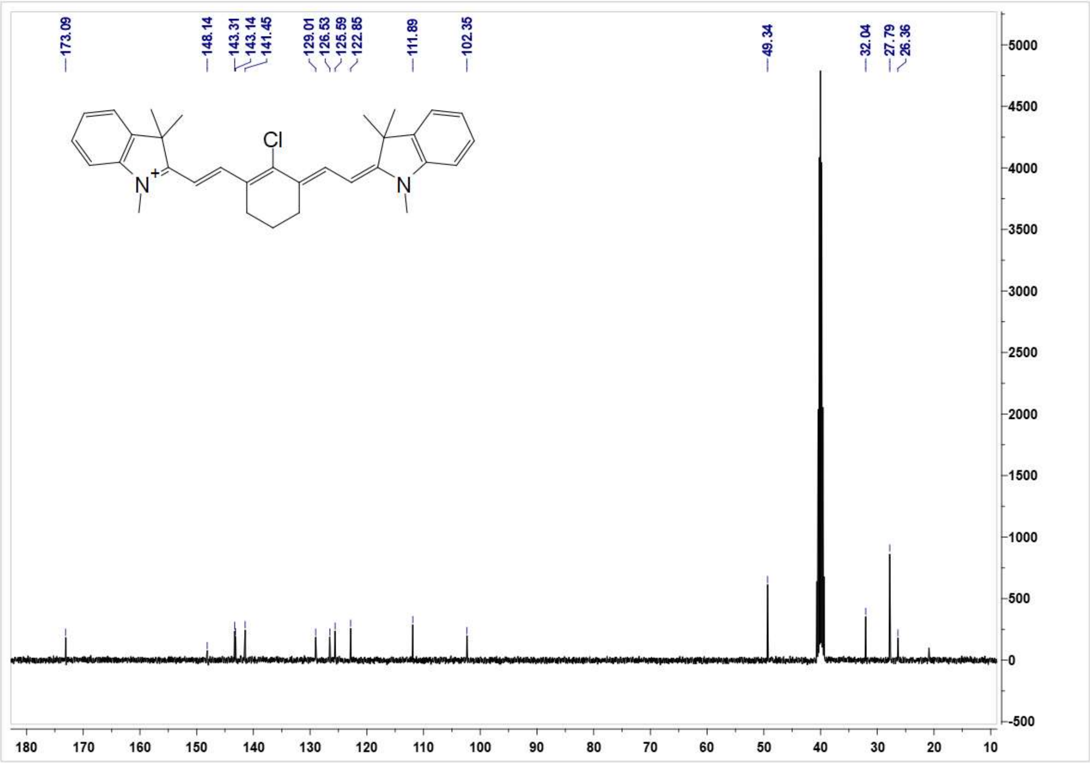
173.09
148.14
143.31
143.14
141.45
129.01
126.53
125.59
122.85
111.89
102.35
49.34
32.04
27.79
26.36
Cl
N+
N

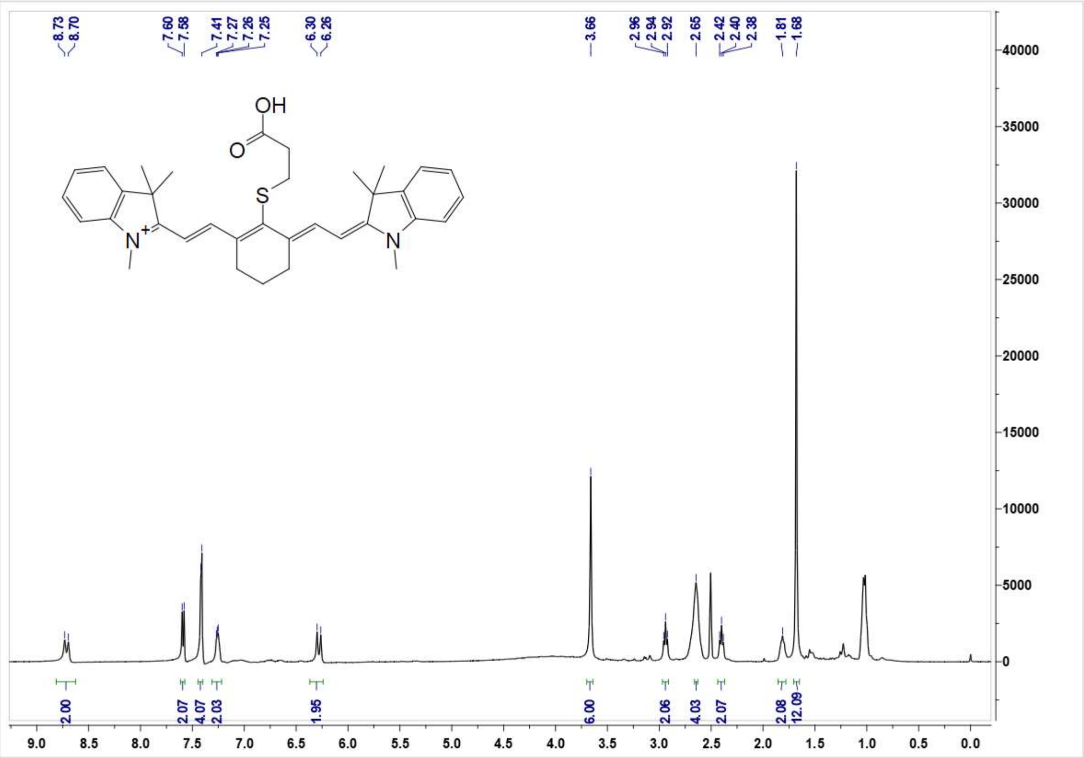

8.73
8.70
7.60
7.58
7.41
7.27
7.26
7.25
6.30
6.26
3.66
2.96
2.94
2.92
2.65
2.42
2.40
2.38
1.81
1.68
OH
O
S
N+
N
2.00
2.07
4.07
2.03
1.95
6.00
2.06
4.03
2.07
2.08
12.09
9.0 8.5 8.0 7.5 7.0 6.5 6.0 5.5 5.0 4.5 4.0 3.5 3.0 2.5 2.0 1.5 1.0 0.5 0.0
40000
35000
30000
25000
20000
15000
10000
5000
0

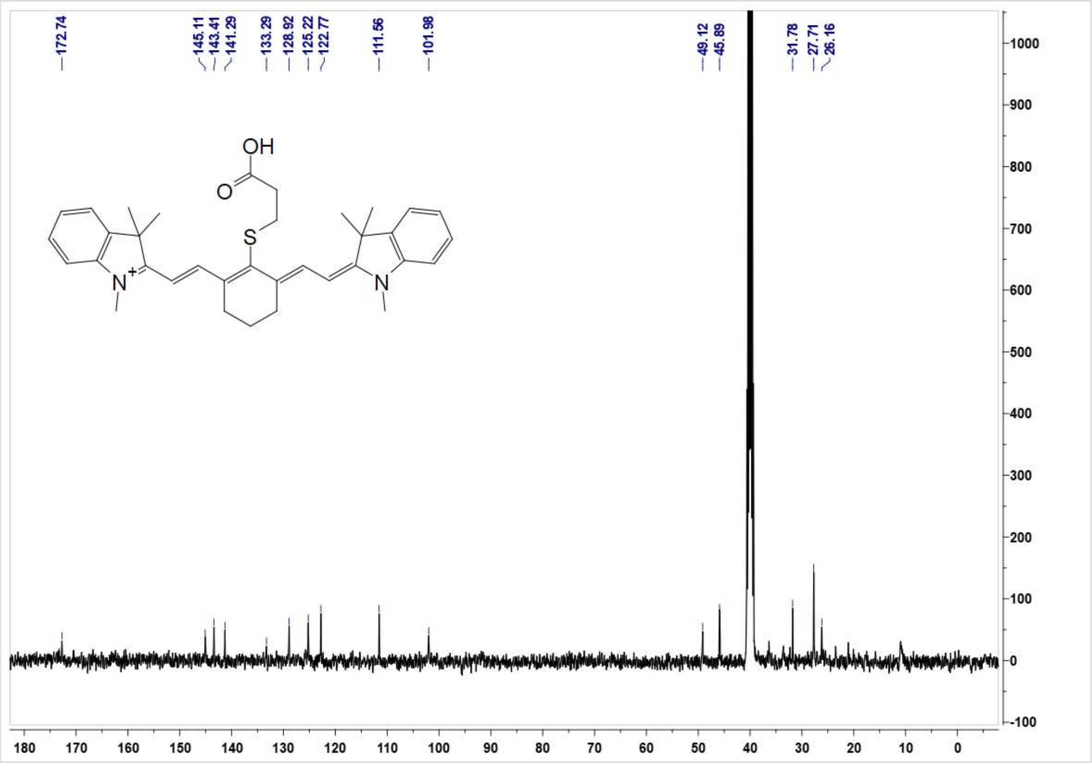
172.74
145.11
143.41
141.29
133.29
128.92
125.22
122.77
111.56
101.98
49.12
45.89
31.78
27.71
26.16
OH
O
S
N+
N
1000
900
800
700
600
500
400
300
200
100
0
-100
180
170
160
150
140
130
120
110
100
90
80
70
60
50
40
30
20
10
0

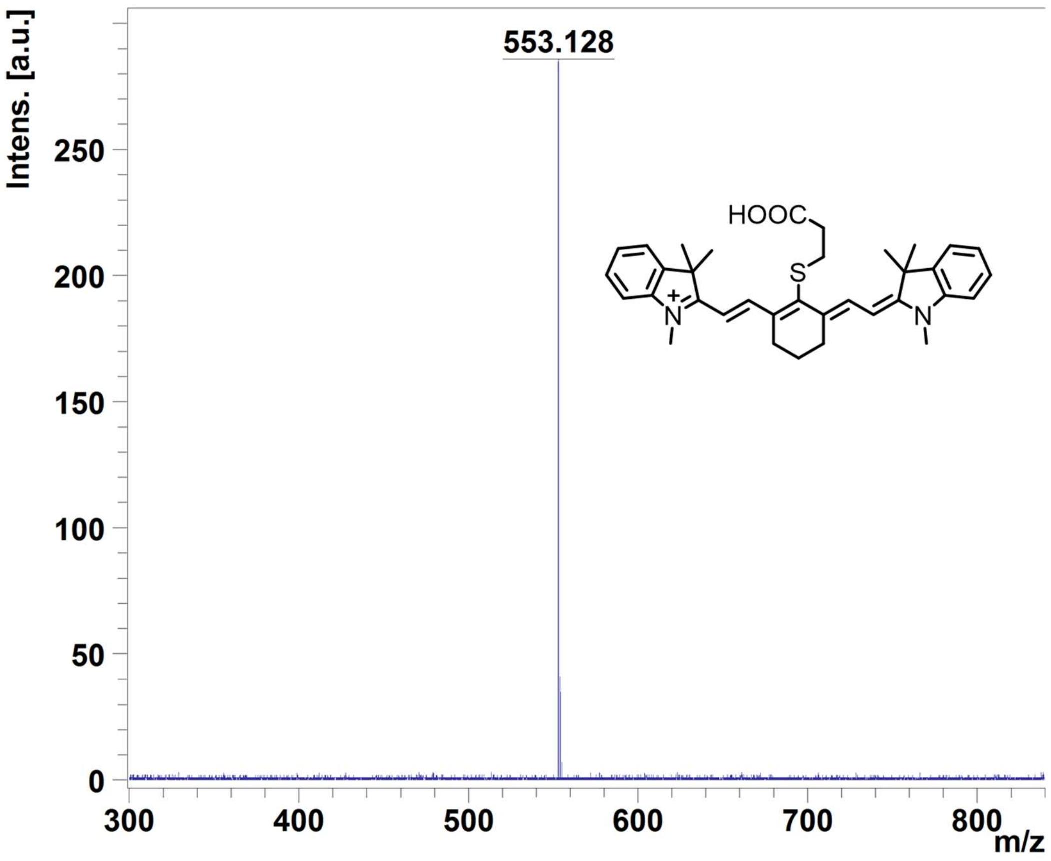
553.128
Intens. [a.u.]
250
200
150
100
50
0
300
400
500
600
700
800
m/z
HOOC
S
N
N

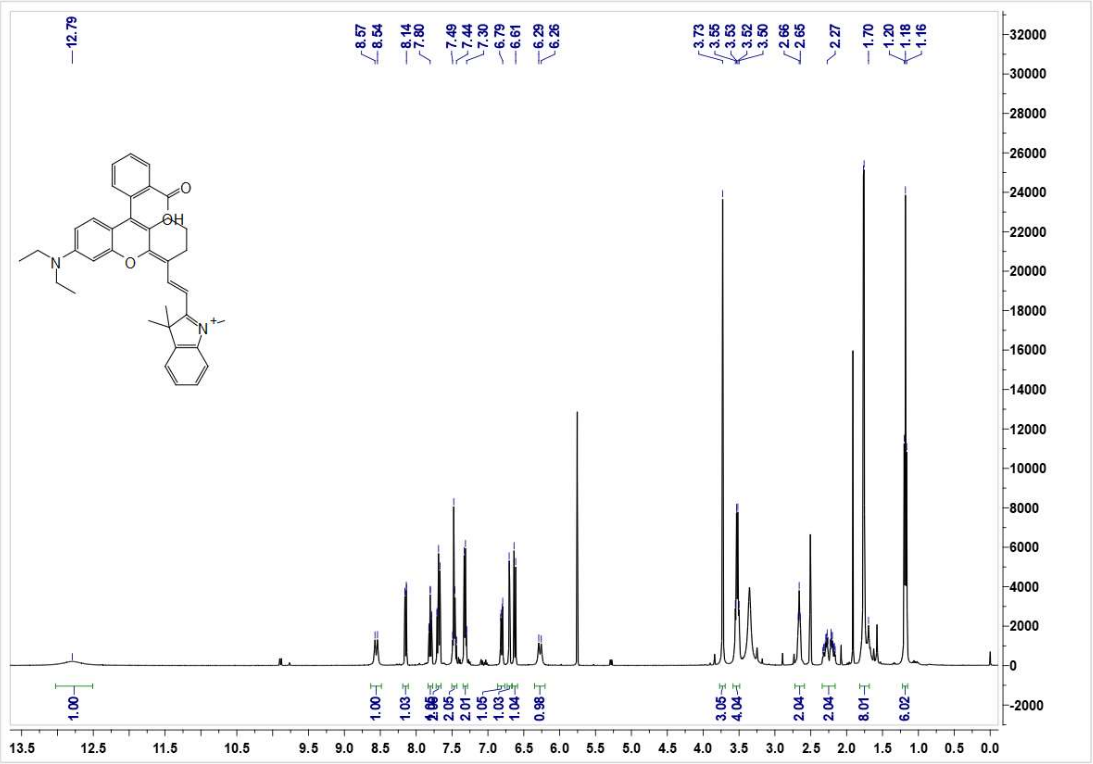

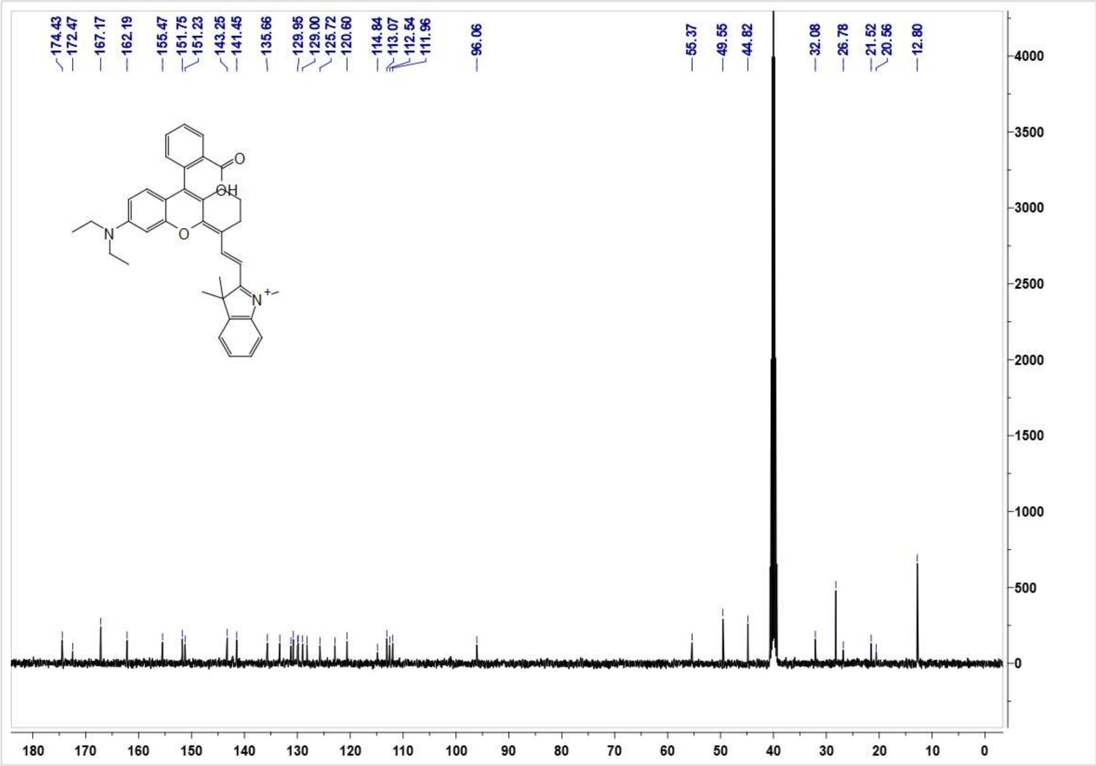
174.43
172.47
167.17
162.19
155.47
151.75
151.23
143.25
141.45
135.66
129.95
129.00
125.72
120.60
114.84
113.07
112.54
111.96
96.06
55.37
49.55
44.82
32.08
26.78
21.52
20.56
12.80
4000
3500
3000
2500
2000
1500
1000
500
0
180 170 160 150 140 130 120 110 100 90 80 70 60 50 40 30 20 10 0
OH
O
N
N+

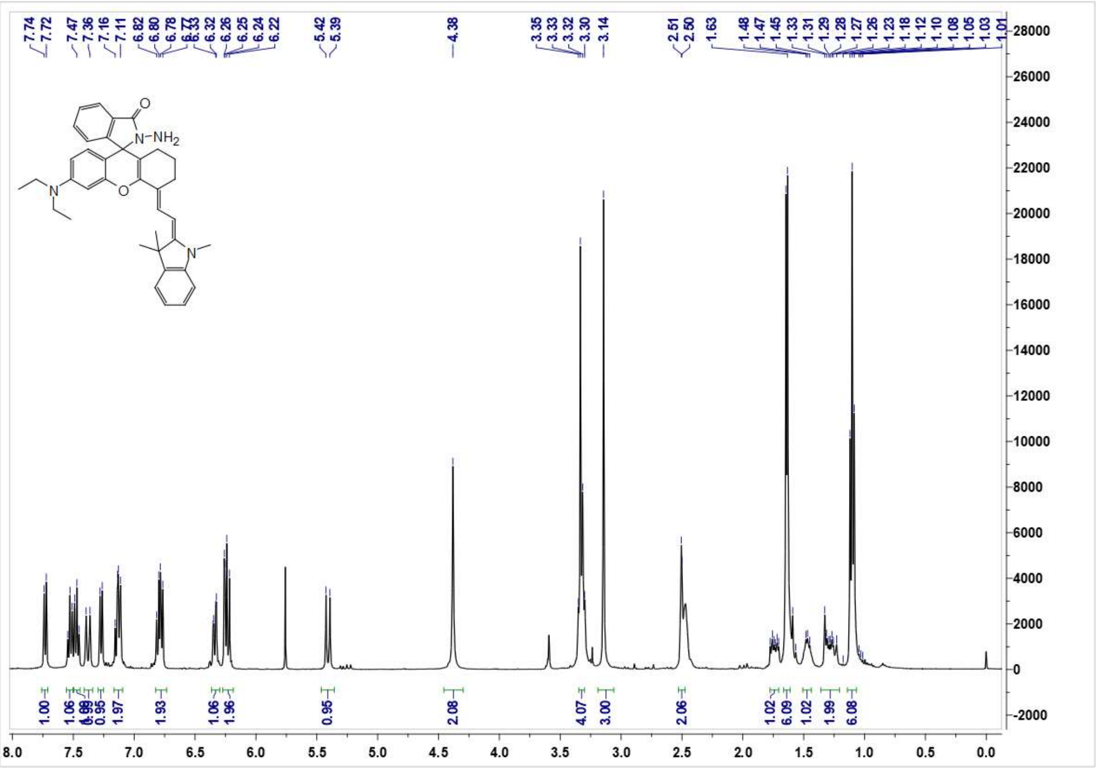
7.74 7.72 7.47 7.36 7.16 7.11 6.82 6.80 6.78 6.77 6.33 6.32 6.26 6.25 6.24 6.22 5.42 5.39 4.38 3.35 3.33 3.32 3.30 3.14 2.51 2.50 1.63 1.48 1.47 1.45 1.33 1.31 1.29 1.28 1.27 1.26 1.23 1.18 1.12 1.10 1.08 1.05 1.03 1.01
1.00 1.06 0.99 0.95 1.97 1.93 1.06 1.96 0.95 2.08 4.07 3.00 2.06 1.02 6.09 1.02 1.99 6.08
8.0 7.5 7.0 6.5 6.0 5.5 5.0 4.5 4.0 3.5 3.0 2.5 2.0 1.5 1.0 0.5 0.0
28000 26000 24000 22000 20000 18000 16000 14000 12000 10000 8000 6000 4000 2000 0 -2000
N-NH2
O
N

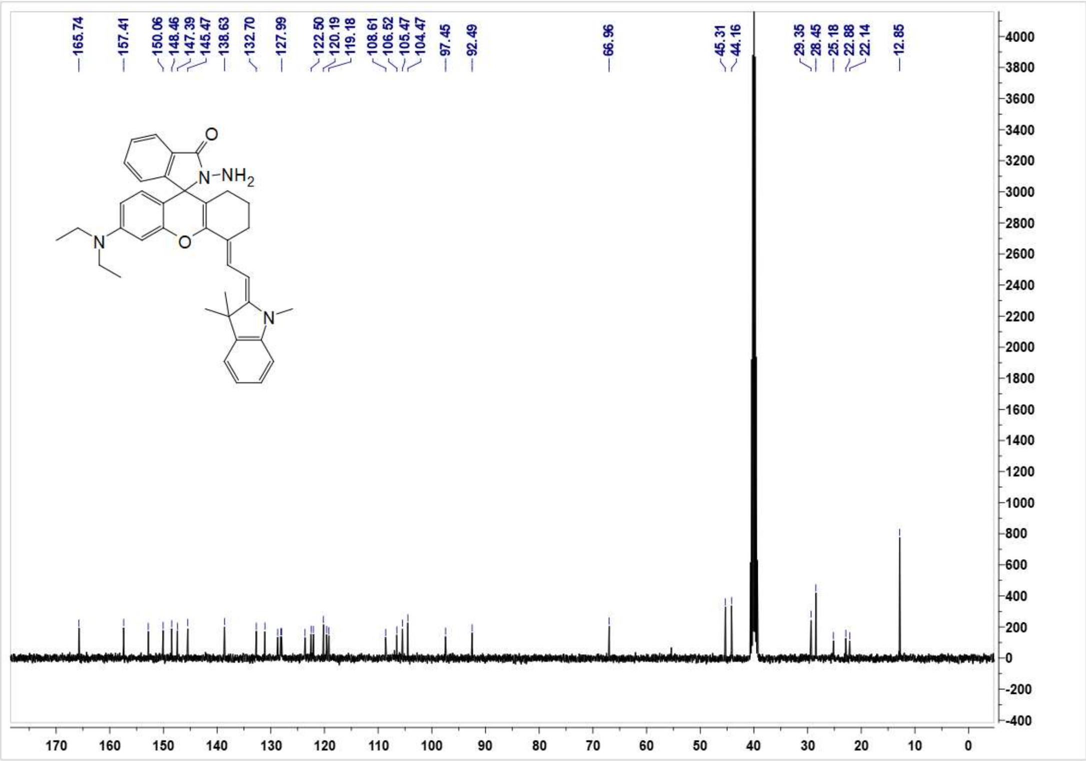
165.74
157.41
150.06
148.46
147.39
145.47
138.63
132.70
127.99
122.50
120.19
119.18
108.61
106.52
105.47
104.47
97.45
92.49
66.96
45.31
44.16
29.35
28.45
25.18
22.88
22.14
12.85
O
N–NH2
N
O
N
170 160 150 140 130 120 110 100 90 80 70 60 50 40 30 20 10 0
4000 3800 3600 3400 3200 3000 2800 2600 2400 2200 2000 1800 1600 1400 1200 1000 800 600 400 200 0 -200 -400

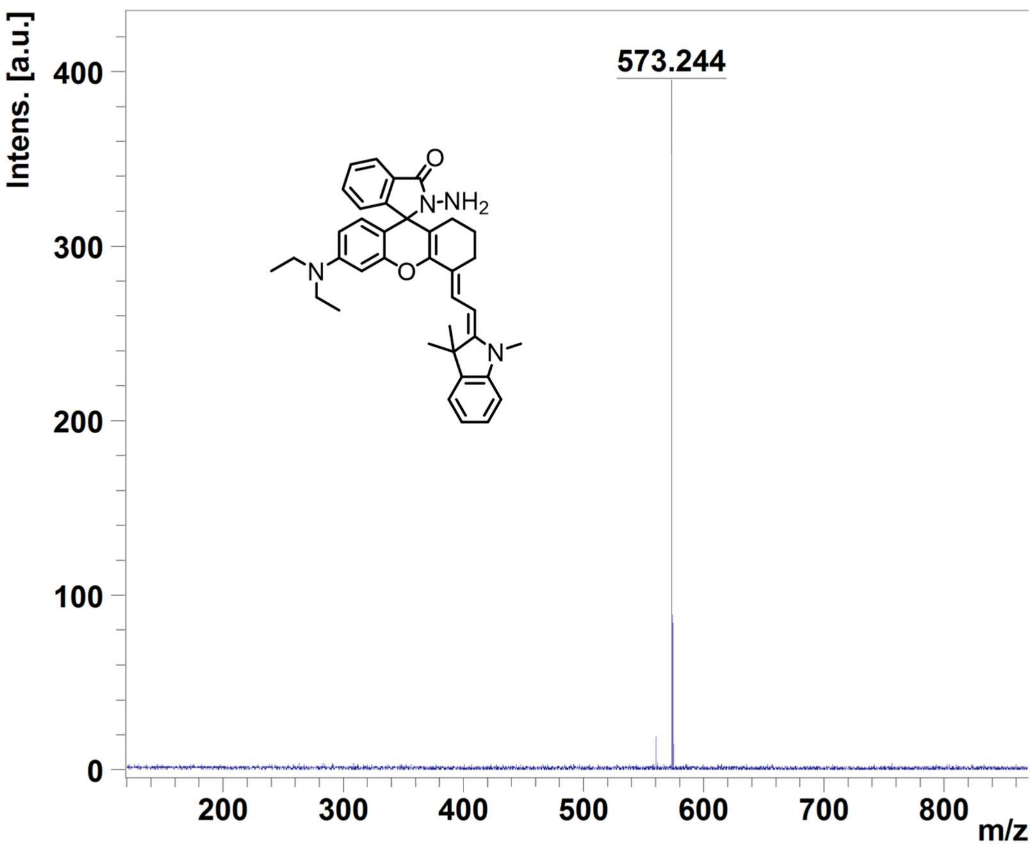
573.244
Intens. [a.u.]
400
300
200
100
0
200
300
400
500
600
700
800
m/z
O
N-NH2
N
O
N

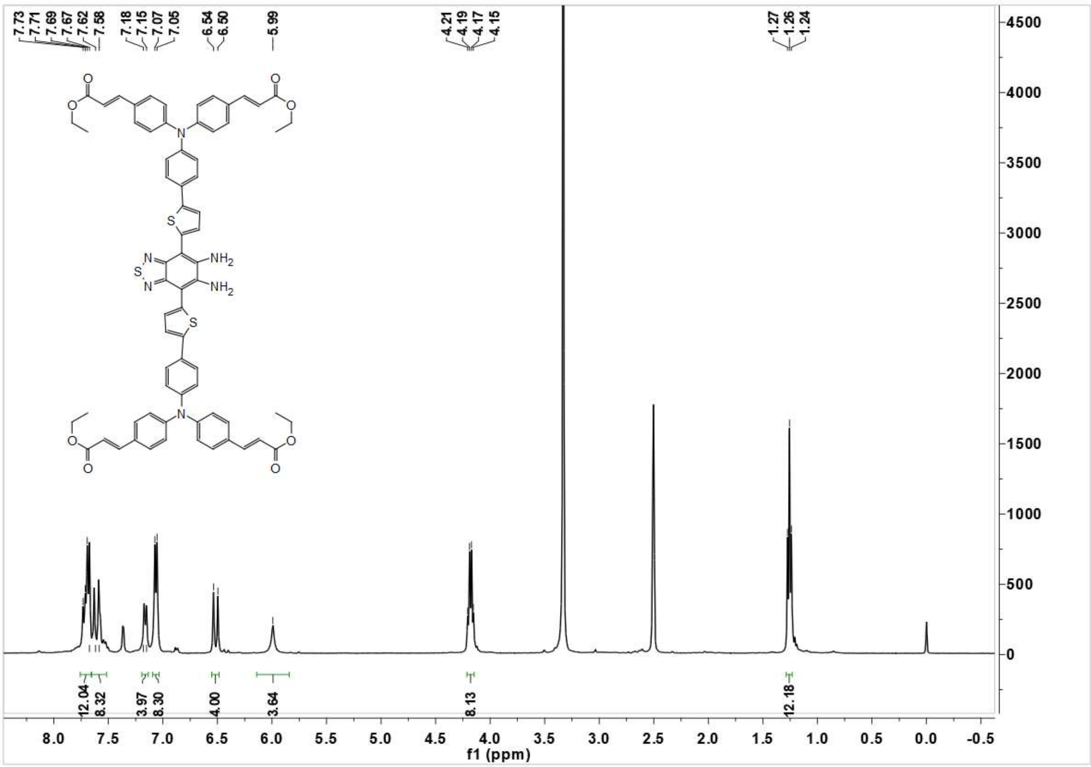
7.73
7.71
7.69
7.67
7.62
7.58
7.18
7.15
7.07
7.05
6.54
6.50
5.99
4.21
4.19
4.17
4.15
1.27
1.26
1.24
NH2
NH2
12.04
8.32
3.97
8.30
4.00
3.64
8.13
12.18
f1 (ppm)

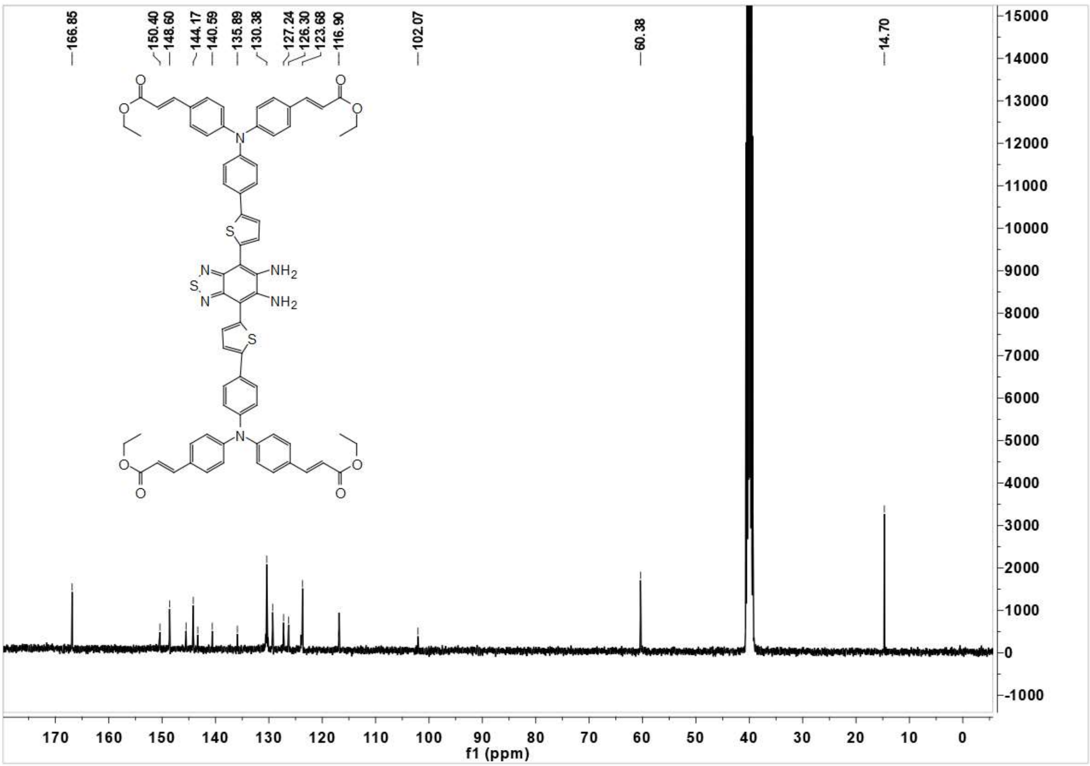
166.85
150.40
148.60
144.17
140.59
135.89
130.38
127.24
126.30
123.68
116.90
102.07
60.38
14.70
NH2
NH2
f1 (ppm)

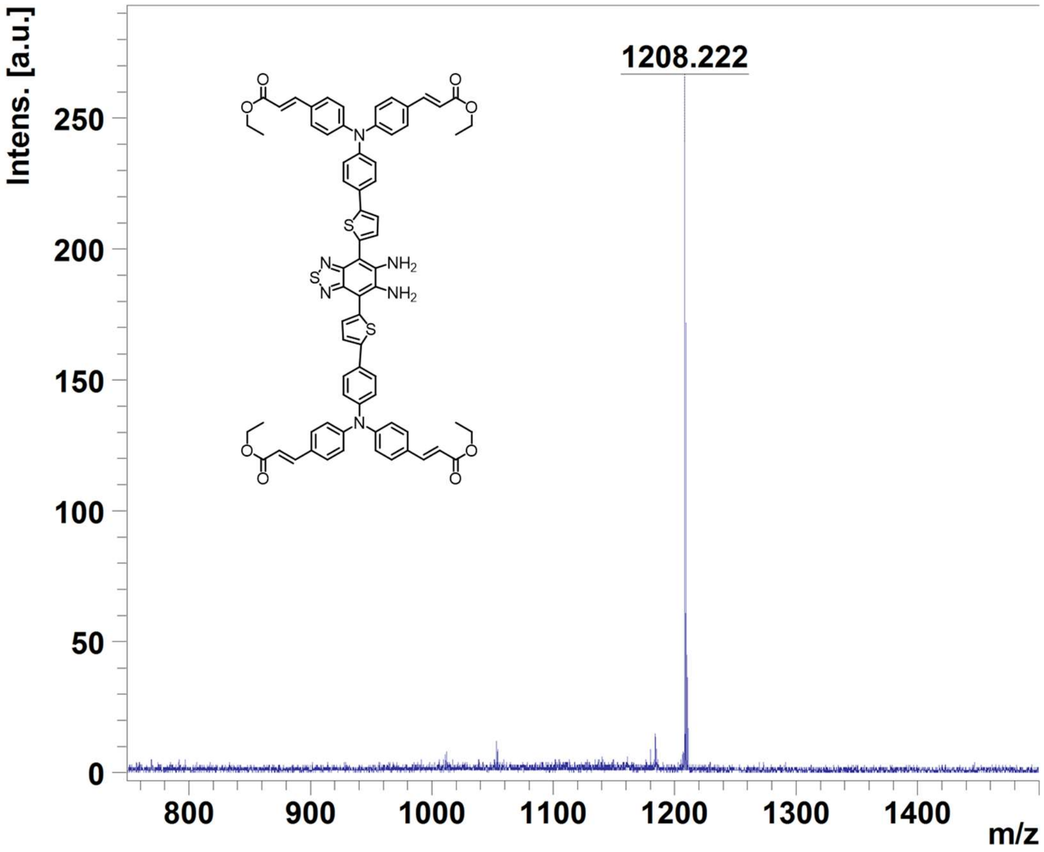

1208.222
Intens. [a.u.]
250
200
150
100
50
0
800
900
1000
1100
1200
1300
1400
m/z
NH2
NH2
N
S
N
S
S
N
N
O
O

References

1. Teng. L. *et al.* Nitric Oxide-Activated "Dual-Key-One-Lock" Nanoprobe for in Vivo Molecular Imaging and High-Specificity Cancer Therapy. *J. Am. Chem. Soc.* **141**, 13572-13581 (2019).
2. Yuan, L., Lin, W., Yang, Y. & Chen, H. A unique class of near-infrared functional fluorescent dyes with carboxylic-acid-modulated fluorescence ON/OFF switching: rational design, synthesis, optical properties, theoretical calculations, and applications for fluorescence imaging in living animals. *J. Am. Chem. Soc.* **134**, 1200-1211 (2012).
3. Peng. J. *et al.* Real-Time In Vivo Hepatotoxicity Monitoring through Chromophore-Conjugated Photon-Upconverting Nanoprobes. *Angew. Chem. Int. Ed.* **56**, 4165-4169 (2017).
4. Almeida-Marrero, V. *et al.* Porphyrinoid biohybrid materials as an emerging toolbox for biomedical light management. *Chem. Soc. Rev.* **47**, 7369-7400 (2018).
5. Zhao, J., Wu, W., Sun, J. & Guo, S. Triplet photosensitizers: from molecular design to applications. *Chem. Soc. Rev.* **42**, 5323-5351 (2013).
6. Zhao, J. *et al.* The triplet excited state of Bodipy: formation, modulation and application. *Chem. Soc. Rev.* **44**, 8904-8939 (2015).
