# Supplementary material for: Ratiometric afterglow luminescent nanoplatform enables reliable quantification and molecular imaging
Source: Nat Commun. 2022 Apr 25;13:2216. doi: 10.1038/s41467-022-29894-1 (PMC9039063; doi:10.1038/s41467-022-29894-1)
Supplement: Supplementary file 2 — Reporting Summary [file 41467_2022_29894_MOESM2_ESM.pdf]

nature portfolio

Corresponding author(s): Xiao-Bing Zhang, Guosheng Song

Last updated by author(s): Mar 1, 2022

Reporting Summary

Nature Portfolio wishes to improve the reproducibility of the work that we publish. This form provides structure for consistency and transparency in reporting. For further information on Nature Portfolio policies, see our Editorial Policies and the Editorial Policy Checklist.

Statistics

For all statistical analyses, confirm that the following items are present in the figure legend, table legend, main text, or Methods section.

n/a	Confirmed	
☐	☒	The exact sample size (*n*) for each experimental group/condition, given as a discrete number and unit of measurement
☐	☒	A statement on whether measurements were taken from distinct samples or whether the same sample was measured repeatedly
☐	☒	The statistical test(s) used AND whether they are one- or two-sided<br>*Only common tests should be described solely by name; describe more complex techniques in the Methods section.*
☒	☐	A description of all covariates tested
☒	☐	A description of any assumptions or corrections, such as tests of normality and adjustment for multiple comparisons
☐	☒	A full description of the statistical parameters including central tendency (e.g. means) or other basic estimates (e.g. regression coefficient) AND variation (e.g. standard deviation) or associated estimates of uncertainty (e.g. confidence intervals)
☐	☒	For null hypothesis testing, the test statistic (e.g. *F*, *t*, *r*) with confidence intervals, effect sizes, degrees of freedom and *P* value noted<br>*Give P values as exact values whenever suitable.*
☒	☐	For Bayesian analysis, information on the choice of priors and Markov chain Monte Carlo settings
☒	☐	For hierarchical and complex designs, identification of the appropriate level for tests and full reporting of outcomes
☒	☐	Estimates of effect sizes (e.g. Cohen's *d*, Pearson's *r*), indicating how they were calculated

Our web collection on statistics for biologists contains articles on many of the points above.

Software and code

Policy information about availability of computer code

Data collection	UV and fluorescence data were obtained by using the Shimadzu UV Probe software and Flsol software. Histological data were obtained by MicroManager. Living Image software was used to obtain fluorescence and afterglow imaging data in the IVIIS imaging system. CaseViewer software was used to obtain H&E staining data.
Data analysis	Imaging data was analyzed using Living Image 4.3 software (PerkinElmer). Confocal fluorescence microscopy imaging data was analyzed using FV10-ASW 2.0 Viewer (Olympus). All statistical calculations were performed using Origin 9.0 (OriginLab). NMR data were analyzed using MestReNova software (Mestre lab Research S.L.).

For manuscripts utilizing custom algorithms or software that are central to the research but not yet described in published literature, software must be made available to editors and reviewers. We strongly encourage code deposition in a community repository (e.g. GitHub). See the Nature Portfolio guidelines for submitting code & software for further information.

Data

Policy information about availability of data

All manuscripts must include a data availability statement. This statement should provide the following information, where applicable:

- Accession codes, unique identifiers, or web links for publicly available datasets
- A description of any restrictions on data availability
- For clinical datasets or third party data, please ensure that the statement adheres to our policy

The data that support the findings of this study are available from the corresponding author on request.

Field-specific reporting

Please select the one below that is the best fit for your research. If you are not sure, read the appropriate sections before making your selection.

- [x] Life sciences
- [ ] Behavioural & social sciences
- [ ] Ecological, evolutionary & environmental sciences

For a reference copy of the document with all sections, see nature.com/documents/nr-reporting-summary-flat.pdf

Life sciences study design

All studies must disclose on these points even when the disclosure is negative.

Sample size	Sample sizes were selected to achieve at least 80% power at a significance level of 0.05. The sample size for each experiment is detailed in the Replication section of Methods.
Data exclusions	No data was excluded from the analysis.
Replication	The experiment was repeated at least three independent experiments with similar results. All experiments have been reproduced to reliably support the conclusions stated in the manuscript.
Randomization	Mice and cells were randomized into different experimental groups.
Blinding	Blinding was not used for other experiments that did not involve biological samples or comparison between different groups, as blinding was not necessary for these experiments and might induce transposition errors.

Reporting for specific materials, systems and methods

We require information from authors about some types of materials, experimental systems and methods used in many studies. Here, indicate whether each material, system or method listed is relevant to your study. If you are not sure if a list item applies to your research, read the appropriate section before selecting a response.

Materials & experimental systems

n/a	Involved in the study	
[ ]	[x]	Antibodies
[ ]	[x]	Eukaryotic cell lines
[x]	[ ]	Palaeontology and archaeology
[ ]	[x]	Animals and other organisms
[x]	[ ]	Human research participants
[x]	[ ]	Clinical data
[x]	[ ]	Dual use research of concern

Methods

n/a	Involved in the study	
[x]	[ ]	ChIP-seq
[ ]	[x]	Flow cytometry
[x]	[ ]	MRI-based neuroimaging

Antibodies

Antibodies used	Primary antibodies: Rabbit anti-iNOS antibody (abcam, ab283655); anti-mouse CD16/32 (BD Biosciences, 553140); mouse anti-CD86-PE (Thermo Fisher Scientific, 12-0862-82); mouse anti-CD80-APC (Thermo Fisher Scientific, 17-0801-82); mouse anti-CD11b-Alexa Fluor 488 (Thermo Fisher Scientific, 53-0112-82); mouse anti-F4/80-PE-Cy5 (Thermo Fisher Scientific, 15-4801-82).<br>Secondary antibodies: Alexa Fluor 488-labeled Goat Anti-Rabbit IgG (Beyotime, A0423).
Validation	All antibodies were used in the research according to the manufacturer's information. Specifically, we relied on the references listed on the manufacture's website:<br>1.https://www.abcam.cn/inos-antibody-rm1017-ab283655.html<br>2.https://www.bdbiosciences.com/zh-cn/products/reagents/flow-cytometry-reagents/research-reagents/single-color-antibodies-ruo/purified-na-le-rat-anti-mouse-cd16-cd32.553140<br>3.https://www.thermofisher.cn/cn/zh/antibody/product/CD86-B7-2-Antibody-clone-GL1-Monoclonal/12-0862-82<br>4.https://www.thermofisher.cn/cn/zh/antibody/product/CD80-B7-1-Antibody-clone-16-10A1-Monoclonal/17-0801-82<br>5.https://www.thermofisher.cn/cn/zh/antibody/product/CD11b-Antibody-clone-M1-70-Monoclonal/53-0112-82<br>6.https://www.thermofisher.cn/cn/zh/antibody/product/F4-80-Antibody-clone-BM8-Monoclonal/15-4801-82<br>7.https://www.beyotime.com/product/A0423.htm

Eukaryotic cell lines

Policy information about cell lines

Cell line source(s)	4T1 cells and RAW264.7 cells were purchased from Cell Bank of Chinese Academy of Sciences.
Authentication	Both 4T1 cells and RAW264.7 cell line were not authenticated.
Mycoplasma contamination	The cell line was not tested for mycoplasma contamination.
Commonly misidentified lines (See ICLAC register)	No ICLAC cell lines were used.

Animals and other organisms

Policy information about studies involving animals; ARRIVE guidelines recommended for reporting animal research

Laboratory animals	6-8 weeks-old female Balb/c mice were purchased from Hunan Slake Jingda Laboratory Animal Co., Ltd. The male Nos2–/– mice (KOCMP-18126-Nos2-B6N-VA) mice were purchased from Cyagen Biosciences (Suzhou) Inc. The animals were group-housed on a 12 h: 12 h light: dark cycle (temperature: 20–25 °C, humidity: 50-65 %) in College of Biology of Hunan University, and fed with food and water ad libitum as appropriate.
Wild animals	The study did not involve animals.
Field-collected samples	This study did not include samples collected from the field.
Ethics oversight	All animal procedures were performed in accordance with the Guidelines for Care and Use of Laboratory Animals of Hunan University, and experiments were approved by the Animal Ethics Committee of the College of Biology (Hunan University).

Note that full information on the approval of the study protocol must also be provided in the manuscript.

Flow Cytometry

Plots

Confirm that:

- [x] The axis labels state the marker and fluorochrome used (e.g. CD4-FITC).
- [x] The axis scales are clearly visible. Include numbers along axes only for bottom left plot of group (a 'group' is an analysis of identical markers).
- [x] All plots are contour plots with outliers or pseudocolor plots.
- [x] A numerical value for number of cells or percentage (with statistics) is provided.

Methodology

Sample preparation	Sample preparation is described in detail in Methods.
Instrument	BD C6 Plus
Software	FlowJo-V10
Cell population abundance	No cell sorting was performed
Gating strategy	Briefly, single cells were selected using forward and side scatter linearity. The expression gating strategy is indicated in Supplementary Fig. 30.

- [x] Tick this box to confirm that a figure exemplifying the gating strategy is provided in the Supplementary Information.
